# Polyimides as Promising Materials for Lithium-Ion Batteries: A Review

**DOI:** 10.1007/s40820-023-01104-7

**Published:** 2023-05-24

**Authors:** Mengyun Zhang, Li Wang, Hong Xu, Youzhi Song, Xiangming He

**Affiliations:** Institute of Nuclear and New Energy Technology, Tsinghua University, Beijing, 100084 People’s Republic of China

**Keywords:** Polyimides, Separators, Binders, Solid-state electrolytes, Lithium-ion batteries

## Abstract

Lithium-ion batteries (LIBs) have helped revolutionize the modern world and are now advancing the alternative energy field. Several technical challenges are associated with LIBs, such as increasing their energy density, improving their safety, and prolonging their lifespan. Pressed by these issues, researchers are striving to find effective solutions and new materials for next-generation LIBs. Polymers play a more and more important role in satisfying the ever-increasing requirements for LIBs. Polyimides (PIs), a special functional polymer, possess unparalleled advantages, such as excellent mechanical strength, extremely high thermal stability, and excellent chemical inertness; they are a promising material for LIBs. Herein, we discuss the current applications of PIs in LIBs, including coatings, separators, binders, solid-state polymer electrolytes, and active storage materials, to improve high-voltage performance, safety, cyclability, flexibility, and sustainability. Existing technical challenges are described, and strategies for solving current issues are proposed. Finally, potential directions for implementing PIs in LIBs are outlined.

## Highlights

- Polyimides (PIs) as coatings, separators, binders, solid-state electrolytes, and active storage materials help toward safe, high-performance, and long-life lithium-ion batteries (LIBs).
- Strategies to design and utilize PI materials have been discussed, and the future development trends of PIs in LIBs are outlooked.

## Graphical abstract

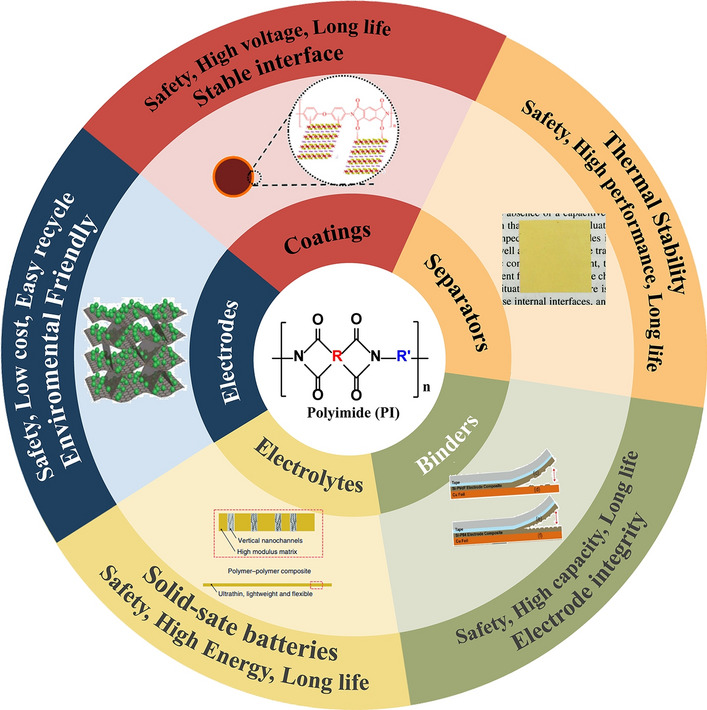

## Introduction

The ongoing energy crisis and environmental issues attributed to traditional energy sources have aroused extensive attention [1, 2]. New energy sources in place of traditional energy sources have become an inevitably developing trend. Lithium-ion batteries (LIBs), as one of the most attractive energy sources, have been commercialized for decades [3–7]. However, several technological challenges such as increasing energy density, improving safety, and extending longevity still urgently need to be overcome. Overview of the rapid development of LIBs in the past 30 years, the critical issues solved and significant technical innovations all benefit from the advancements of state-of-the-art materials [8, 9]. The era of new materials has arrived.

LIBs are composed of the cathode, anode, separator, electrolyte, and coin shells involving various constituent materials incorporation of organic materials, inorganic materials, and metals. Particularly, polymers, as one kind of organic material, are not extensively used in LIBs, besides the separator and binder. However, with the continued improvement of researchers’ cognitions, more polymeric components are now being considered for LIBs [10, 11], including different functional polymer coatings, the current popular solid-state polymer electrolytes, new polymer active storage materials, etc. Nowadays, two dilemmas of polymers applied in LIBs should be dealt with. One is that several technique and usage problems faced by traditional polymer components limit their practical applications, which should be replaced by new-style polymer materials. For example, traditional polyolefin separators cannot withstand high temperatures, which replaced by high-performance separators is encouraged. The other one is using new polymer components to boost LIBs. One compelling case is employing functional polymer coatings to protect key components from deteriorations of high voltage or interfacial side reactions. Additionally, new styles of solid-state polymer electrolytes and polymer active storage materials are also developing trends. In a word, polymer materials are playing more and more important roles in LIBs.

**Fig. 1:**
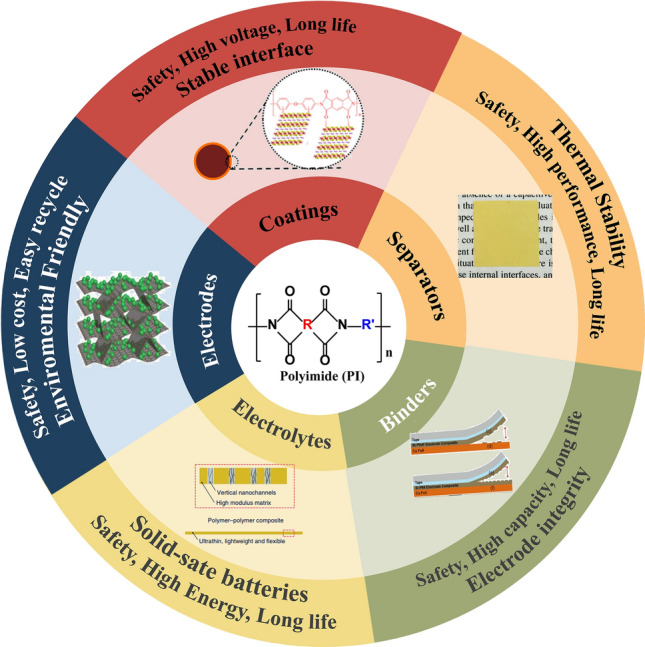
Schematic showing how polyimides (PIs) are promising polymer materials for LIBs. As coatings, PIs can stabilize the interface of the cathode and electrolyte. PI separators can enhance thermal stability, while PI binders can maintain the structural integrity of electrodes. PIs can increase the energy density of LIBs if employed as solid-state electrolyte support. PIs can replace traditional inorganic active storage materials. All these promising PI applications help toward safe, high-performance, and long-life LIBs

LIBs propose very high-performance requirements for polymer materials, including excellent chemical resistance, thermal stability, high-voltage tolerance, and even high mechanical strength, while polyimides (PIs) are standing out among many polymers. PIs can be used as coatings [12], binders [13], separators [14], solid-state electrolytes [15], active storage materials [16], etc. Though the applications of PIs are diverse, they are still confined to the laboratory and far away from commercialization. PIs are formed from anhydride and amine polycondensations [17]. PIs have been in development since their introduction in 1955. There are two methods for synthesizing PIs: One is the hydrothermal method, also called the one-step method, and the other is the two-step method, which includes thermal imine and chemical imine methods [18]. The hydrothermal method involves direct polymerization in a high boiling point solvent. The high temperatures and pressures involved in the procedure can be a safety concern. The chemical imine method refers to PI precursors (polyamic acid, PAA) dehydrated and cyclized with the aid of a catalyst. Compared with these two methods, the thermal imine method involves PAA cyclization at high temperatures without introducing other substances, confirming to be a more economical and convenient way. PIs can be categorized into aliphatic and aromatic. Generally, aliphatic PIs are flexible and soluble, mainly used as coatings or binders. In contrast, aromatic PIs are rigid and insoluble, mainly used as membranes or solid powders. Table 1 lists the important physical and chemical properties of different PI applications in LIBs. PIs are promising in LIBs. PIs can be coated onto active material surfaces to keep the cathode and electrolyte interface stable at high-voltage conditions, increasing the cyclability of the cell. PIs can also be used as separators to improve safety when operating at high temperatures. To prolong the battery life, researchers can employ PI binders to enhance the electrode structural integrity. PIs can also be employed in solid-state lithium batteries. Furthermore, PIs can replace traditional energy storage materials to lower the cost and environmental pollution. Figure 1 outlines the requirements of each LIB component that can be satisfied by incorporating PIs into LIBs.

**Table 1:** Physical and chemical properties of different PI applications in LIBs

PIs in LIBs	Solubility	Critical properties	Preparation methods
PI coatings	Insoluble	Good electrolyte wettability	Self-growing and solvothermal methods
PI separators	Insoluble	Thermally stable membrane	Template, phase separation, and electrospinning methods
PI binders	Soluble	Soluble and adhesive	Chemical synthesis
Solid PI electrolytes	Soluble	Soluble and lithium-ion conductive	Chemical synthesis
Solid PI-based electrolytes	Insoluble	Membrane with porous holes	Etching, casting
PI active storage materials	Insoluble	Reversible lithium-ion storage ability at special voltage conditions and powdery	Chemical synthesis

From PI materials to LIBs, much effort should be devoted to solving various issues encountered. Herein, we summarize the important issues involving PIs in LIBs. We describe the essential features for each issue encountered when employing PIs. Moreover, we address several critical technology challenges and strategies for using PIs in LIBs. Finally, future development directions for LIBs with PIs are outlined.

## PIs in Different LIB Components

### Improving High-Voltage Performance: PI Coatings on Cathode Materials

Energy storage devices with high energy and power densities for portable electric devices, electric vehicles, and grid energy storage are being investigated intensively [19]. To increase the energy density of LIBs, researchers have two strategies: increase the specific capacity or increase the operating voltage according to Eq. 1:

1 $$E = C_{0} \times V$$

where $E$ represents the energy density, $C_{0}$ is the theoretical specific capacity, and $V$ is the average operating voltage. For cathode materials, much effort has been devoted to finding materials that operate below 4.35 V but exhibit high specific capacity, i.e., Ni-rich LiMO_2_ (M = Co, Mn, Ni) [20–23]. However, the practical specific capacity of these materials is nearing the limit of ~ 200 mAh g^−1^. Therefore, researchers began to increase the specific capacity by elevating the operating voltage.

When LIBs are at high-voltage conditions, the delithiated cathode and liquid electrolyte promote violent interfacial side reactions, which is the most significant challenge researchers face. Surface coating is an effective technique that reduces interfacial side reactions, thereby enhancing electrochemical performance. To date, many metal compounds, such as Al_2_O_3_ [24–27], ZrO_2_ [28], MoO_3_ [29], TiO_2_ [30], AlF_3_ [31], and Li_3_PO_4_ [32], have been used to coat active materials. Because these metal compounds facilitate Li-ion conductivity and decrease the violent side reactions, irreversible lithium loss is reduced and cycling performance is increased. However, metal oxides face several technical issues, including discontinuity, brittleness, and delamination from the surface of cathode materials. Therefore, selecting polymers with tunable molecular structures and high adhesion as coatings can be an effective strategy.

**Fig. 2:**
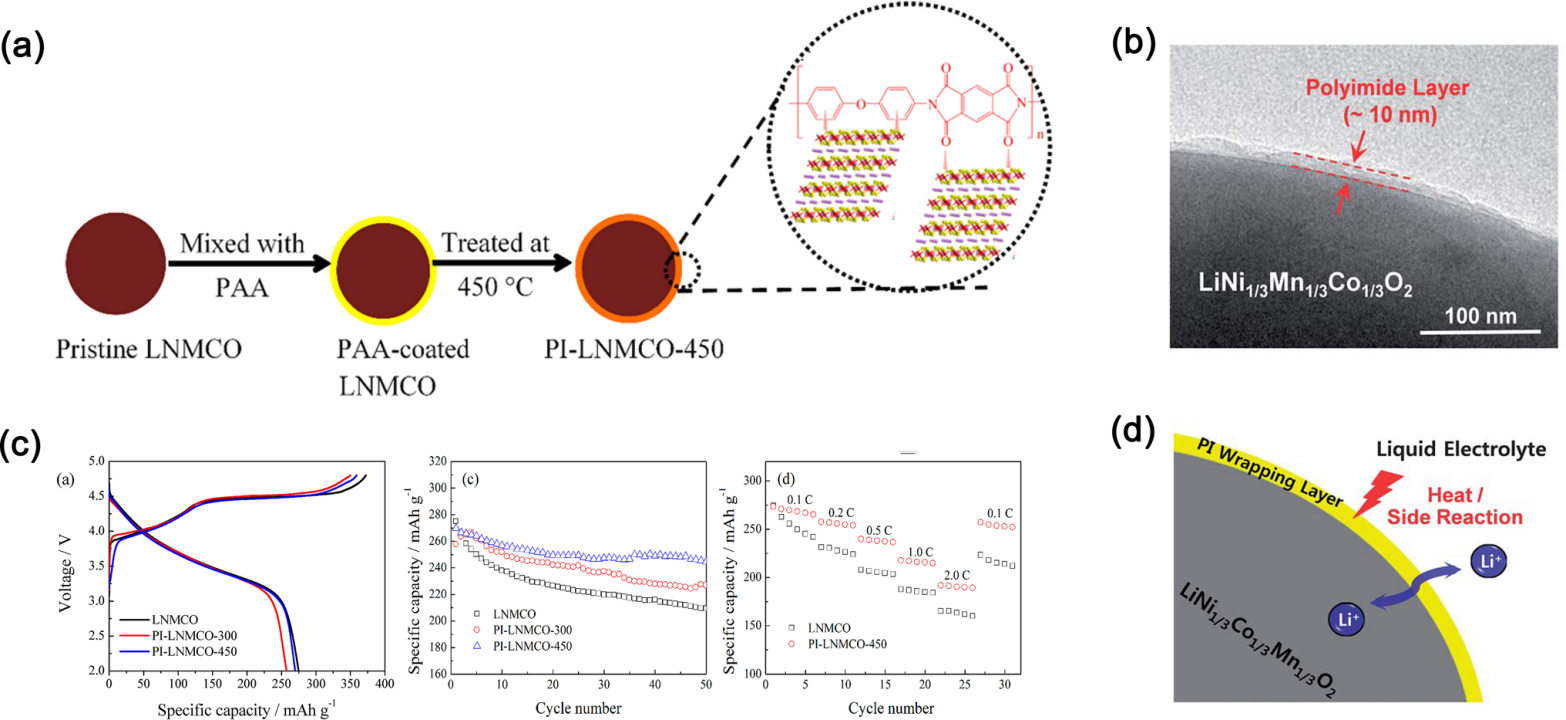
**a** Schematic of PI coating onto an LNMCO cathode material. **b** TEM image of PI-coated LiNi_1/3_Mn_1/3_Co_1/3_O_2_ (PI@NCM333). **c** Electrochemical performances of LNMCO, PI@LNMCO-300, and PI@LNMCO-450, including initial efficiency, cycling, and rate capability (300 and 450 represent thermal imidization temperatures). **d** Schematic of the mechanism for enhanced electrochemical performances in PI-coated NCM333

Among the polymers, PIs with excellent performances such as good elasticity, thermal stability, and electrolyte wettability can be used as ideal coating materials [33, 34]. Especially as coatings, PIs should be insoluble in electrolytes as well as possess good electrolyte wettability. This performance requirement is important for enhancing the electrochemical performance. The synthesis of PI-coated cathode materials is the focus of this section, and the coating process is shown in Fig. 2a. Herein, the thermal imine method is used to realize the coating. First, pyromellitic dianhydride (PMDA) and 4,4′-oxydianiline (ODA) are polymerized to obtain PAA at low temperatures. Then, a certain concentration of PAA solution is mixed with cathode active materials to coat the PAA onto the surface of active material particles. Thereafter, the PAA-coated cathode materials are thermally imidized through a step-by-step heating method to convert PAA to PI coatings [35]. Finally, active material particles with PI nano-coatings are obtained.

Transmission electron microscopy (TEM) is the most straightforward way for evaluating PIs successfully coating onto the surface of cathode particles. Figure 2b is a TEM image of PI-coated LiNi_1/3_Co_1/3_Mn_1/3_O_2_ (NCM333). The coating was continuous and uniform (~ 10 nm thick) [33]. The electrochemical performance of the cell was mainly affected by this PI layer, as shown in Fig. 2c. As seen in this figure, the cyclability of PI-LNMCO (Li_1.2_Ni_0.13_Mn_0.54_Co_0.13_O_2_) is better than that of LNMCO, especially at high thermal imine temperatures. Zhang et al. [35] found that high thermal imidization temperatures facilitate strong interactions between PI coatings and cathode particle surfaces, prohibiting the solvation of transition metal ions and enhancing the electrochemical performance. Figure 2d illustrates the PI-coating mechanism [33]. PI effectively blocks the electrolyte, hindering interfacial side reactions. Meanwhile, Li-ion conductivity in PIs is good [36]. The cathodes with PI coatings exhibit a lower polarization and less irreversible Li-ion loss than uncoated cathodes, resulting in a good cycling lifetime. Additionally, N has special interactions with Mn^4+^, further increasing the rate capability. Currently, the discussion focus is the stability of PI coatings at high voltage. Meanwhile, our group has devoted much work to verifying this question. Our following work concludes that PI coatings are stable at high voltages up to 4.6 V without structural changes. PI coatings play the physical barrier role in retarding the interfacial side reactions. The optimal PI-coating content is about 4wt% (PAA added in the preparation process). As a consequence, PI coatings provide an effective way to improve electrochemical performances at high-voltage conditions.

### Improving Battery Safety: PI Separators

The LIB separator is a porous and insulating membrane located between the cathode and anode, not only providing a pathway for Li-ion conduction but also playing the role of avoiding internal short circuits (ISCs) [37, 38]. Higher demands such as the proper pore size, uniform pore distribution, low pore tortuosity, low heat shrinkage, excellent mechanical performance, and good electrolyte wettability should have for separators [39]. Therefore, the materials chosen and processing technologies for the preparation of good-performance separators are strict.

Commercial separators are made of polyethylene (PE) or polypropylene (PP) owing to their high chemical stability and acceptable mechanical strength. Currently, the processing technologies for polyolefin separators are very mature. The thickness of polyolefin separators can be reduced to less than 15 μm without sacrificing their mechanical property, and the electrochemical performance can be obtained stably at room temperatures. However, two shortcomings of these separators need to be addressed [40]. First, the wettability of polyolefin separators is insufficient in accommodating any electrolyte, resulting in limited ionic conductivity owing to their hydrophobic nature. Second, the high thermal shrinkage and low melting temperature of polyolefin separators easily induce ISCs when operating at high temperatures, leading to thermal runaway. Compared with polyolefin separators, PI membranes exhibit a higher tensile strength of more than 10 Mpa, higher thermal stability up to 500 °C with little shrinkage, and good electrolyte uptake of larger than 100%. Shi et al. [41] have found that the above performances of PI separators are all better than those of PE separators. He et al. [42] employed different structural monomers to synthesize diverse PI separators, and all performance metrics are higher than those of a PP separator. These findings demonstrate that PI separators are preferable separator candidates.

**Fig. 3:**
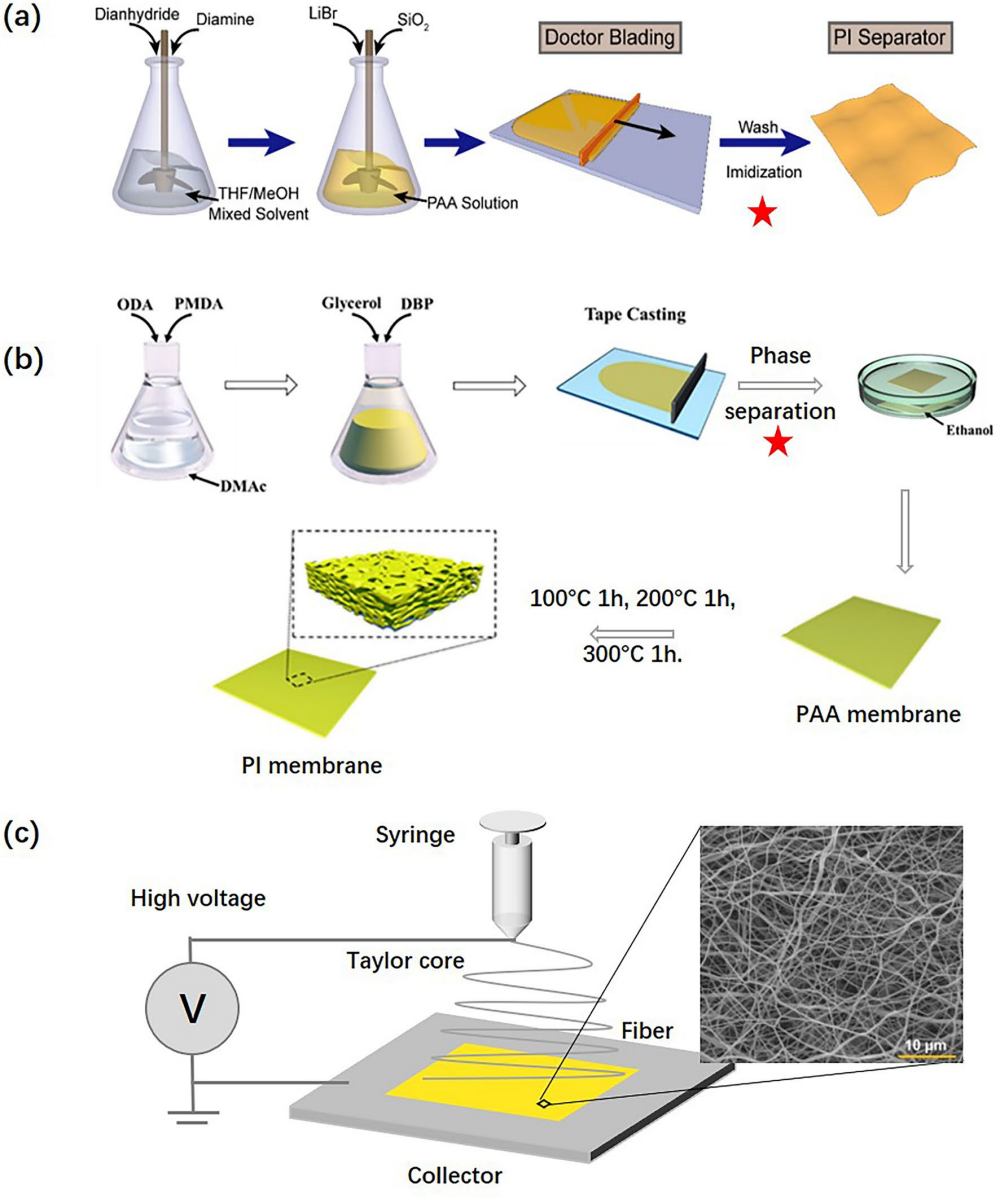
Different preparation methods for PI separators. **a** Template method. **b** Phase separation method. **c** Electrospinning method

PI separators are not yet commercialized due to their complex processing technology and expensive cost. To date, the preparation methods for PI separators are still confined to the laboratory, including template, phase separation, and electrospinning methods. Figure 3a shows a schematic of the template method reported by Lin et al. [43]. Typically, the template method employs inorganic salts as templates to make pores. In their work, LiBr and SiO_2_ were mixed into a PAA solution and doctor blade to produce the membrane. Then, the PAA membrane was thermally imidized to convert it into a PI membrane. Finally, a nanoporous PI membrane was obtained by washing LiBr away with water. The main advantage of this method is that it maintains the intact pore structure. Moreover, the added SiO_2_ enhances electrolyte wettability even further and provides good thermal stability and mechanical properties to the separator, which could widen the service temperature and prohibit Li dendrites effectively. Li et al. [44] reported the phase separation method to fabricate PI separators. As shown schematically in Fig. 3b, dibutyl-phthalate and glycerin are added to the PAA solution, which is then blade onto a glass plate to obtain the PAA membrane. This PAA membrane is further immersed in an ethanol coagulation bath at 40 °C several times to remove the solvent and additives for making holes. Finally, the same method of thermal imidization is adopted to convert the PAA membrane into a PI membrane. The cell assembled with this PI separator exhibited good electrochemical performances at 140 °C, whereas the cell with a PE separator failed. The reason for the good performance of the cell at high temperatures is attributed to the PI separator maintaining good thermal stability without observable shrinkage at high temperatures. Similarly, PI separators prepared by the modified method have been further explored by Song and Zhou et al. [45, 46] to boost the safety of LIBs. Compared with the above methods, the electrospinning method is simpler and more versatile for membrane preparation. Electrospinning is one of the most promising methods to realize PI separator commercialization. Li et al. [47] employed the solution blow spinning for fabricating PI separators. The technique includes three steps: ①synthesizing PAA, ② electrospinning, and ③ thermal imidization. The preparation process is shown in Fig. 3c. As the PI separator made by the electrospinning method possesses a conductive pore structure, it further enhances the ionic conductivity. While the size of the hole is large, the formed Li dendrites easily penetrate the separator and lead to the ISCs. Therefore, many references have reported the modified electrospinning PI separators.

**Fig. 4:**
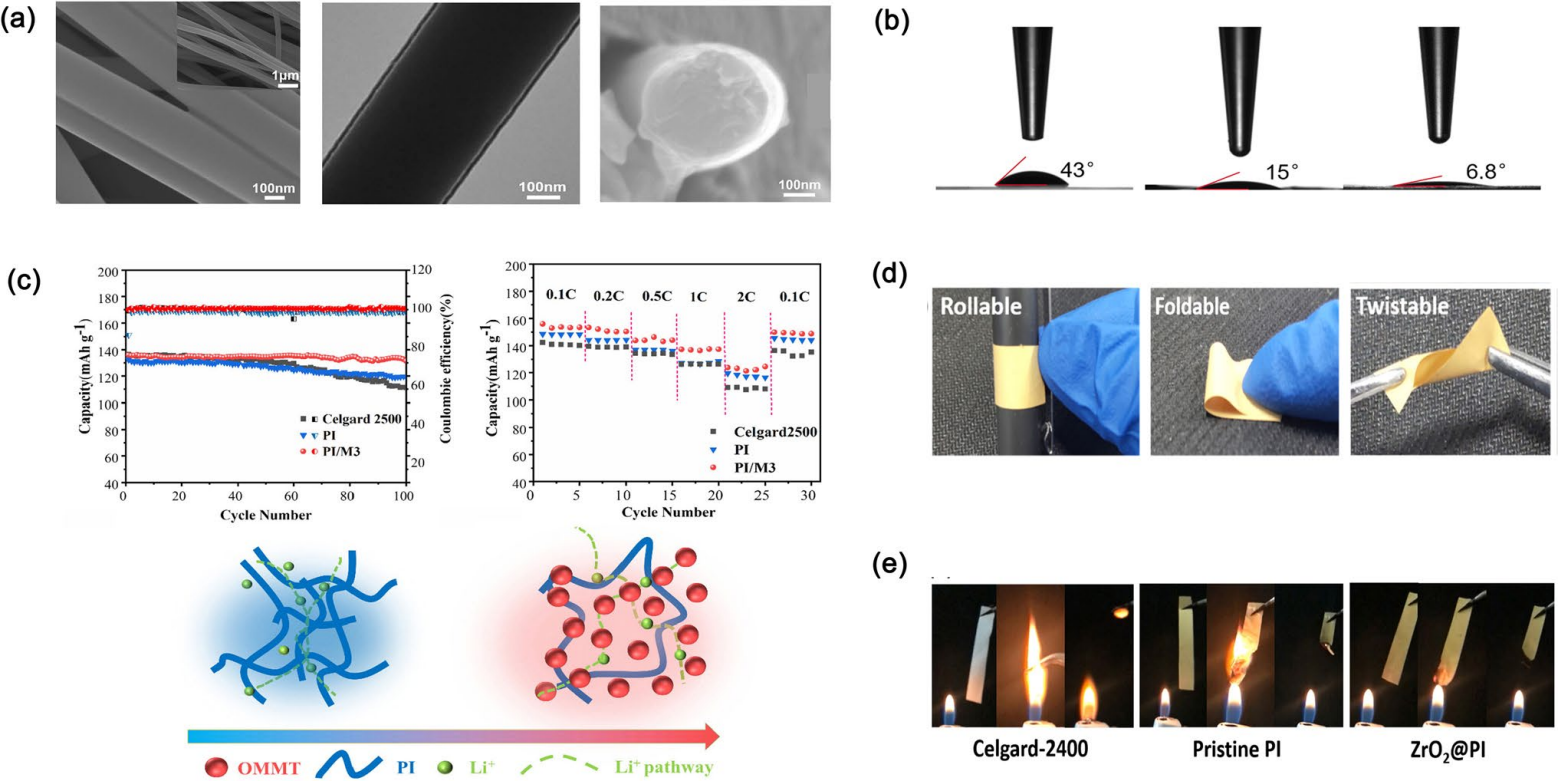
**a** SEM images of PI/SiO_2_ nanofibers. **b** Static contact angles of Celgard-2400, pure PI, and PI/SiO_2_ separators (43°, 15°, and 6.8°, respectively). **c** Cycling and rate capability performances of Celgard-2500, PI, and PI/OMMT separators. **d** Images of the PI/SiO_2_ separator demonstrating its flexibility. **e** Flame retardancy of Celgard-2400, pure PI, and PI/SiO_2_ separators

Pore structure modifications include coating with polymers or inorganic ceramic particles. A polymer coating could reduce pore size to prevent Li dendrites and improve mechanical properties and thermal stability. Besides, inorganic ceramic particle coatings are also beneficial for enhancing electrochemical performances. Currently, much work has been done for PI separators coated with inorganic ceramic materials combined with the electrospinning method [48–50]. Figure 4a illustrates a PI nanofiber separator coated with SiO_2_ particles using the in situ nanoencapsulation hydrolysis method, as reported by Wu et al. [48]. SEM images clearly show that SiO_2_ coatings are uniformly wrapped onto the surfaces of PI nanofibers. The static contact angle of the carbonate-based electrolyte on the PI/SiO_2_ separator was 6.8° as shown in Fig. 4b, lower than that on Celgard and pure PI separators, indicating good electrolyte wettability of the PI/SiO_2_ separator. Qiao et al. [50] fabricated another PI separator reinforced with intercalated organic montmorillonite (OMMT) via solution blow spinning. In Fig. 4c, the cell with the as-prepared PI/OMMT composite separator exhibits better cycling performance (a discharge specific capacity of 131 mAh g^−1^ after 100 cycles at  C) and rate capability (124 mAh g^−1^ at 2 C) than the one with Celgard-2500 separator (107 mAh g^−1^ after 100 cycles at 1 C and 107 mAh g^−1^ at 2 C). This is due to that the electrolyte wettability of the PI/OMMT composite separator is enhanced, which forms the complete Li-ion conductive route and induces higher Li-ion conductivity, resulting in the enhancement of electrochemical performances. The ceramic-coated PI separators also have good mechanical flexibility and flame-retardant properties. Figure 4d shows that PI separators are rollable, foldable, and twistable, guaranteeing good use reliability [49]. Figure 4e shows the comparison of the combustion performances of Celgard-2400, pure PI, and PI/ZrO_2_ separators. The PI/ZrO_2_ separator exhibits the best flame-retardant property, suggesting that incorporating inorganic ceramic particles is beneficial in improving the safety of LIBs [49].

### Improving Cyclability of Structurally Integral Anodes: PI Binders

Graphite dominates the anode market for many years owing to its long cycle life, abundance, and low cost. However, the graphite anode material possesses a relatively lower energy density (375 mAh g^−1^). Therefore, developing alternative anode materials is essential. Silicon is considered to be a promising anode material for next-generation LIBs owing to its abundance and high theoretical specific capacity (4,200 mAh g^−1^). The main challenge in silicon anodes is their extensive volume change (up to 300%) during lithium insertion and extraction, which often leads to the pulverization of active alloy particles and poor cyclability. Nano-silicon [51, 52] and Si/C composite [53–55] can mitigate volume change in silicon. Using suitable binders [56–61] could also prevent silicon particle pulverization. Although the amount of binders in electrodes is little, they influence the performance of silicon-based anodes significantly.

**Fig. 5:**
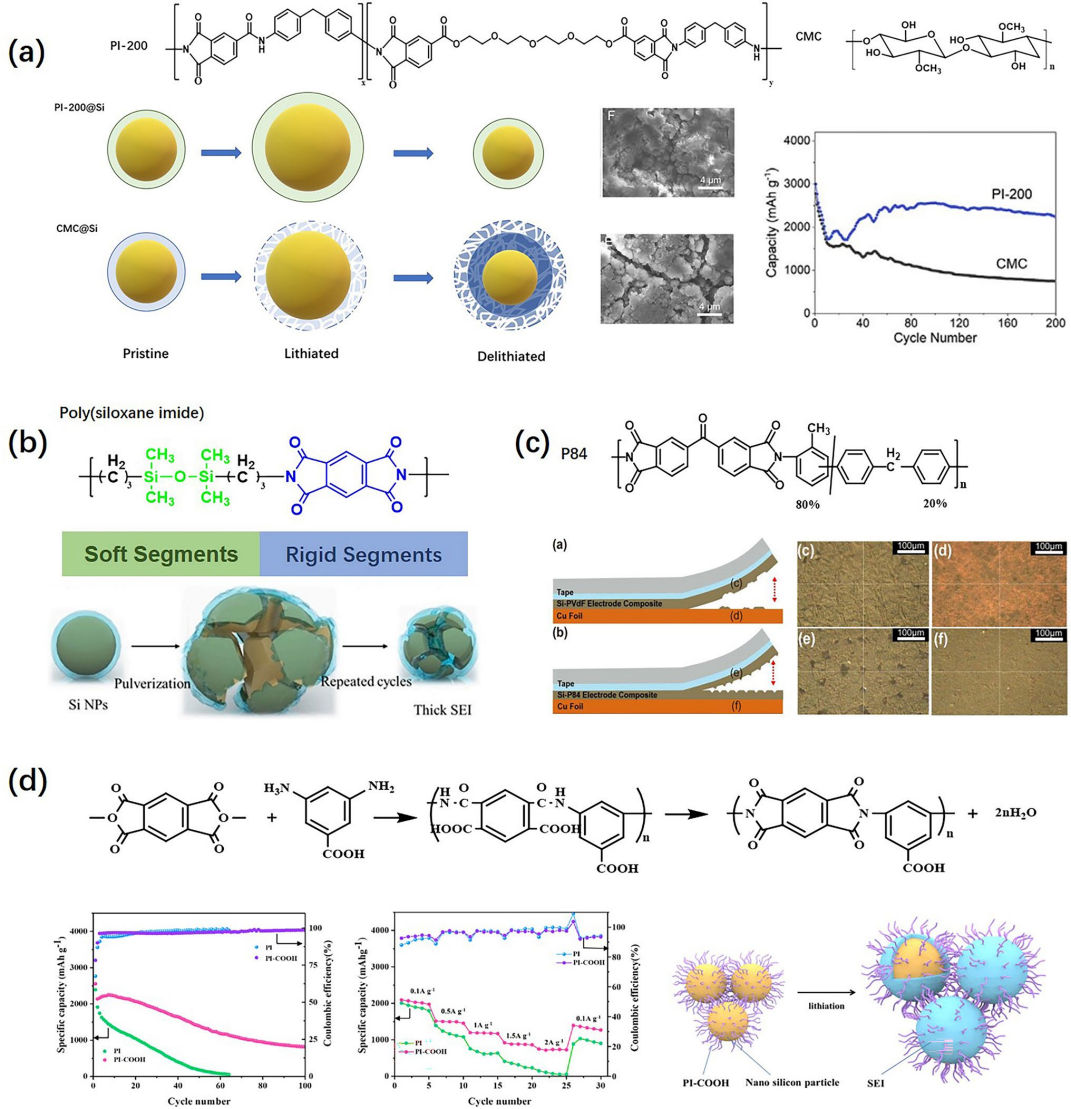
**a** PI-200 synthesis by polymerizing polyethylene glycol (PEG), trimellitic anhydride chloride (TMAC), and 4,4′-methylenedianiline (MDA). Cycling performances, SEM images after cycling, and schematics of lithiation/delithiation comparing PI-200 and CMC binders are also shown. **b** Alternative PI binder design combining soft and rigid segments. **c** P84 vs. PVDF binder in silicon anodes. Images from peeling experiments reveal adhesion effects. **d** The synthesis process of PI-COOH binder, cycling and rate capability, and SEI formations

Various polymer binders, such as the mussel-inspired binder [62–64], polyacrylic acid [58, 65], polysaccharides [66, 67], polyacrylonitrile [68], polyimides [69–71], self-healing polymer binders [61, 72], and conductive binders [73, 74], have been developed. Among these, PI binders with superior adhering properties, good mechanical strength, remarkable thermal stability, and outstanding electrolyte affinity are attracting wide attention. Much work reports PI binders from the perspective of molecular structure design, aiming at ameliorating different properties. To increase the Li-ion conductivity, Yao et al. [75] introduced ether functional group abundant polyethylene glycol (PEG) segments to PI structure. Compared with the carboxymethyl cellulose (CMC) binder, the discharge capacity of the silicon anode using this PI binder is as high as 2,989.7 g^−1^ and with few microcracks generated after cycling shown in Fig. 5a. To depress the pulverization of silicon anode, the soft and rigid copolymerized PI binder was designed [76]. The soft siloxane components possess excellent elasticity to accommodate volume changes. The rigid blocks provide a high modulus to withstand the mechanical stress generated in the silicon anode. The structural diagram of poly(siloxane imide) (PSI) binder is shown in Fig. 5b. To increase the adhesive property, PI binders with stronger adhesive functional groups were synthesized. Choi et al. [77] used highly adhesive and thermally stable co-polyimide (P84) as the silicon anode binder. In Fig. 5c, P84-based electrode was peeled off from the Cu current collector with little residues, while the surface of the Cu current collector was smooth after the PVDF-based electrode was peeled off, suggesting better adhesion with P84 than with PVDF. Besides, high adhesive capability of PI binders could keep the electrode with structural integrity, leading to the good cycling life and rate capability. Xu et al. [78] designed a kind of PI binder with the functional groups of carboxyls tethered laterally shown in Fig. 5d. Due to the interaction between carboxyls and the surface oxide layer of silicon particles, it prohibits the regenerations of solid electrolyte interface (SEI), enhancing the electrochemical performance. To sum up, the reasonable structural design of PI binders endows silicon anodes with better structural integrity and longer cycling lifespan, which lays a good foundation for the commercialization of silicon anodes.

**Fig. 6:**
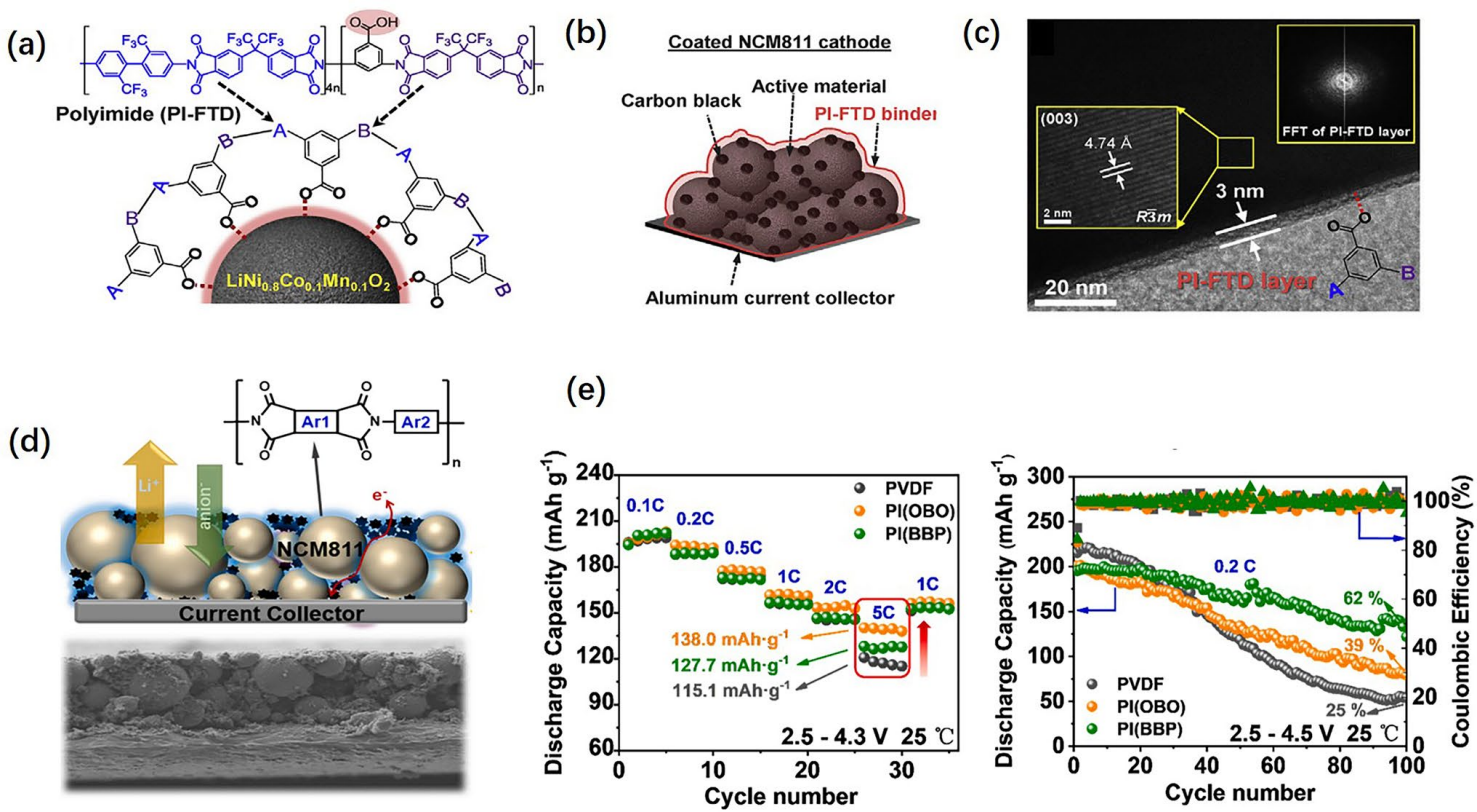
**a, b** Structure schematics of PI-FTD@NCM811 and cathode piece. **c** TEM image for PI-FTD@NCM811. **d** SEM image of the cathode sheet and its corresponding schematic diagram. **e** Rate capability and cycling performance

As PI materials are stable at high voltage, it concludes that PI binders are also stable at high voltage. Combining the merits of good structural integrity and high-voltage resistance, PI binders can be applied in cathodes. Similarly, the design of PI binders for cathodes is purposive. Pham et al. [79] designed a block copolymerized PI binder containing –CF_3_ and –COOH. Due to the –COOH, this PI binder will form strong interactions with LiNi_0.8_Co_0.1_Mn_0.1_O_2_ (NCM811), whose structure is shown in Fig. 6a. Due to the –CF_3,_ it endows the PI binder with good thermal stability and chemical inertness. The structure of the electrode piece is shown in Fig. 6b, and the thickness of the binder layer is about 3 nm shown in Fig. 6c. Qi et al. [80] also designed two kinds of PI cathode binders, namely PI(BBP) and PI(OBO). These two PI binders were, respectively, mixed with active materials LiNi_0.8_Co_0.1_Mn_0.1_O_2_ (NCM811) and conducting materials super-P in the ratio of 8:1:1 to obtain cathode sheet, whose cross-sectional SEM image and the corresponding schematic diagram are shown in Fig. 6d. The CR2032 coin cells assembled with these two kinds of cathodes show good rate capability and cycling performance in Fig. 6e. No matter PI binders are used for cathodes or silicon anodes, the electrode structural integrity can be assured, leading to excellent electrochemical performances.

### Application in All-Solid-State Lithium-Ion Batteries: Solid PI-based Electrolytes

To realize all-solid-state (ASS) lithium-ion batteries (LIBs) with higher safety and higher energy density, solid-state electrolytes (SSEs) are pivotal as they play a significant role in determining comprehensive electrochemical performance [81, 82]. SSEs should be highly ionically conductive, mechanically strong, chemically/electrochemically stable, nonvolatile, and non-flammable. SSEs can be classified into three types: inorganic (ceramic/glass) solid electrolytes (ISEs), solid polymer electrolytes (SPEs), and composite solid-state electrolytes (CSEs) [83]. Though ISEs possess excellent Li-ion conductivity, the intrinsic brittleness and easy pulverization of ISEs affect their use reliability. SPEs present continuous and flexible features, while SPEs face a big challenge in the improvement of Li-ion conductivity. Combining both advantages of ISEs and SPEs, CSEs show great development potential in SSEs.

For preparing SPEs even CSEs, choosing a suitable polymer matrix is the first consideration. Poly(ethylene oxide) (PEO) has become the most widely used electrolyte material owing to its high solubility toward lithium salts and relatively high Li-ion conductivity. However, PEO-style SSEs do not prohibit Li dendrites and are not stable at high temperatures. PIs possess excellent mechanical strength, good electrolyte wettability [84], and outstanding thermal stability, suggesting that they can be a good candidate for the SSE matrix. Generally, PI SSEs could be categorized as solid PI and solid PI-based electrolytes. Solid PI electrolytes are synthesized by introducing PEO molecular segments to PI main chains, while the solid PI-based electrolytes are synthesized by introducing PEO electrolytes to a PI porous membrane. Currently, only a few studies have employed PI directly as an electrolyte [85]. Much work has been reported on solid PI-based electrolytes. Furthermore, solid PI-based electrolytes are divided into solid PI-based SPEs and solid PI-based CSEs.

**Fig. 7:**
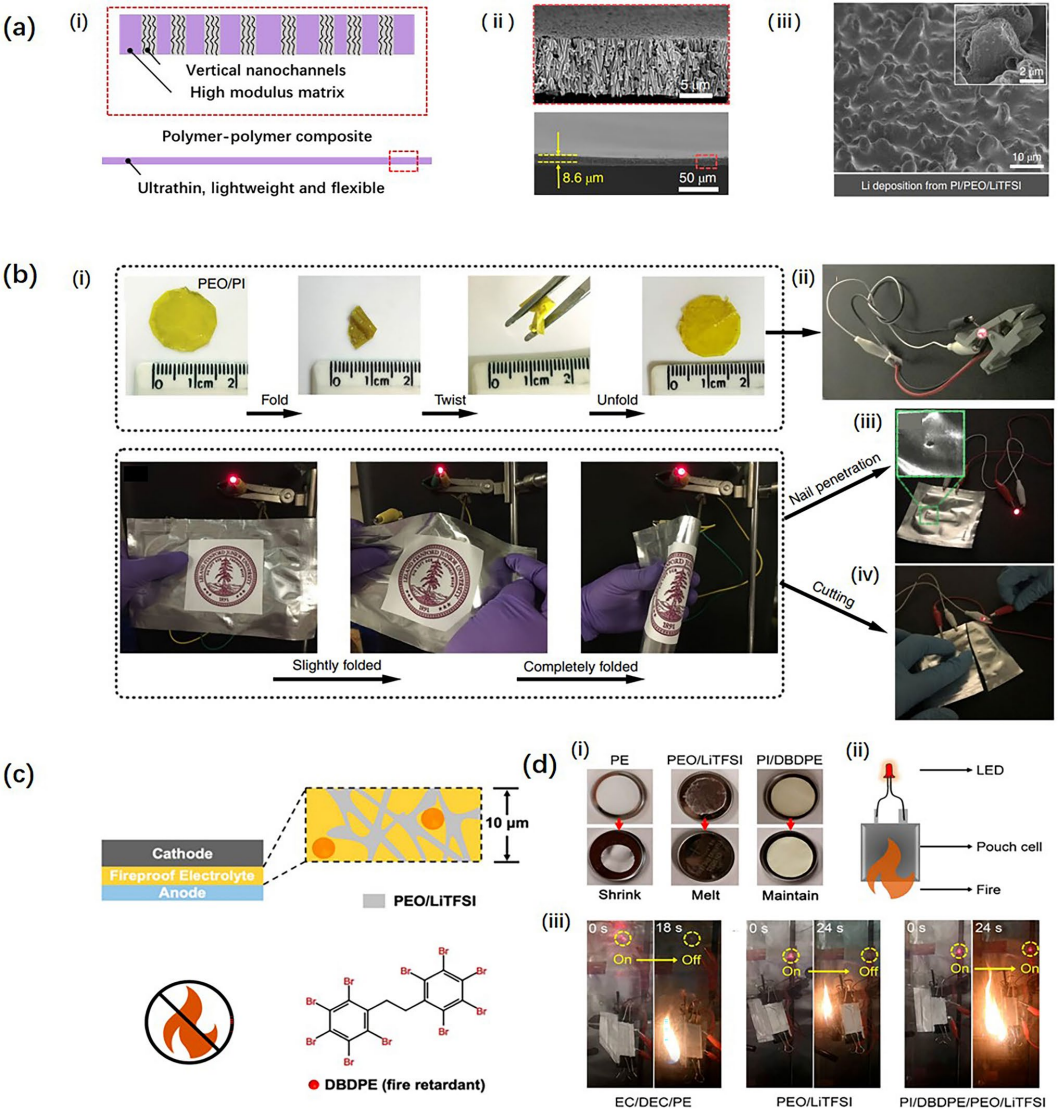
**a** Vertical porous nanochannels made using the track-etching method on a PI film (i), obtaining ultrathin, lightweight, and flexible PI solid polymer electrolytes (ii). A uniform Li deposition layer was observed by SEM after cycling (iii). **b** Abuse tests for PI/PEO/LiTFSI-based LIBs: (i) folding performance; (ii) button batter lighting an LED bulb; (iii) flexible Li/PI/PEO/LiTFSI/LFP pouch cell lighting an LED bulb; and (iv, v) nail and cutting tests. **c** Flame-retardant PI SPE by adding the fire-retardant DBDPE. **d** Thermal abuse tests: (i) Photographs of the PE separator, PEO/LiTFSI, and PI/DBDPE film before and after exposure to thermal shock (150 °C, 0.5 h); (ii) schematic illustration of the thermal abuse test of the pouch cell battery; and (iii) flame abuse test of batteries with EC/DEC/PE, PEO/LiTFSI, and PI/DBDPE/PEO/LiTFSI as electrolytes

For solid PI-based SPEs, Wan et al. [86] fabricated a kind of ultrathin and flexible solid PI-based electrolytes, as shown in Fig. 7a(i). Aligned channels were made on the thin Kapton PI film by using the track-etching method. Then PEO/lithium bis-(trifluoromethanesulfonyl)imide (LiTFSI)/acetonitrile solution was dropped and spun on this porous PI membrane to infiltrate the pores. SEM images for the cross-sections of the PI SPE before and after cycling are shown in Fig. 7a(ii). The thickness of the PI SPE is ~ 8 μm. The electrode surface is smooth even after cycling, demonstrating Li dendrites were effectively prohibited by the PI SPE, as shown in Fig. 7a(iii). Additionally, the PI SPE-assembled pouch cell can still operate under folding, twisting, and unfolding shown in Fig. 7b(i, ii) and the pouch cell can still light an LED bulb after the nail and cutting tests shown in Fig. 7b(iii, iv). To further increase the safety, Cui et al. [87] designed a flame-retardant PI SPE with a fire-retardant additive, decabromodiphenyl ethane (DBDPE) cast on the PI matrix, whose structure is shown schematically in Fig. 7c. In Fig. 7d(i), the PE, PEO/LiTFSI, and PI/DBDPE membranes exhibit different thermal behaviors under high-temperature conditions. Due to the different melting points of the PE, PEO, and PI matrix, PE shrank, PEO melted, while PI can withstand high temperatures and maintain its original morphology. In extreme cases shown in Fig. 7d(ii, iii), the PE membrane-assembled pouch cell can no longer function when burned. The LED bulb was off after 18 s. Under the same conditions, the pouch cell assembled with the PEO/LiTFSI SPE no longer functions after 24 s. However, the pouch cell assembled with the PI/DBDPE/PEO/LiTFSI SPE still functions for more than 24 s. The excellent work shows that the PI SPE has a great commercial prospect for safety.

**Fig. 8:**
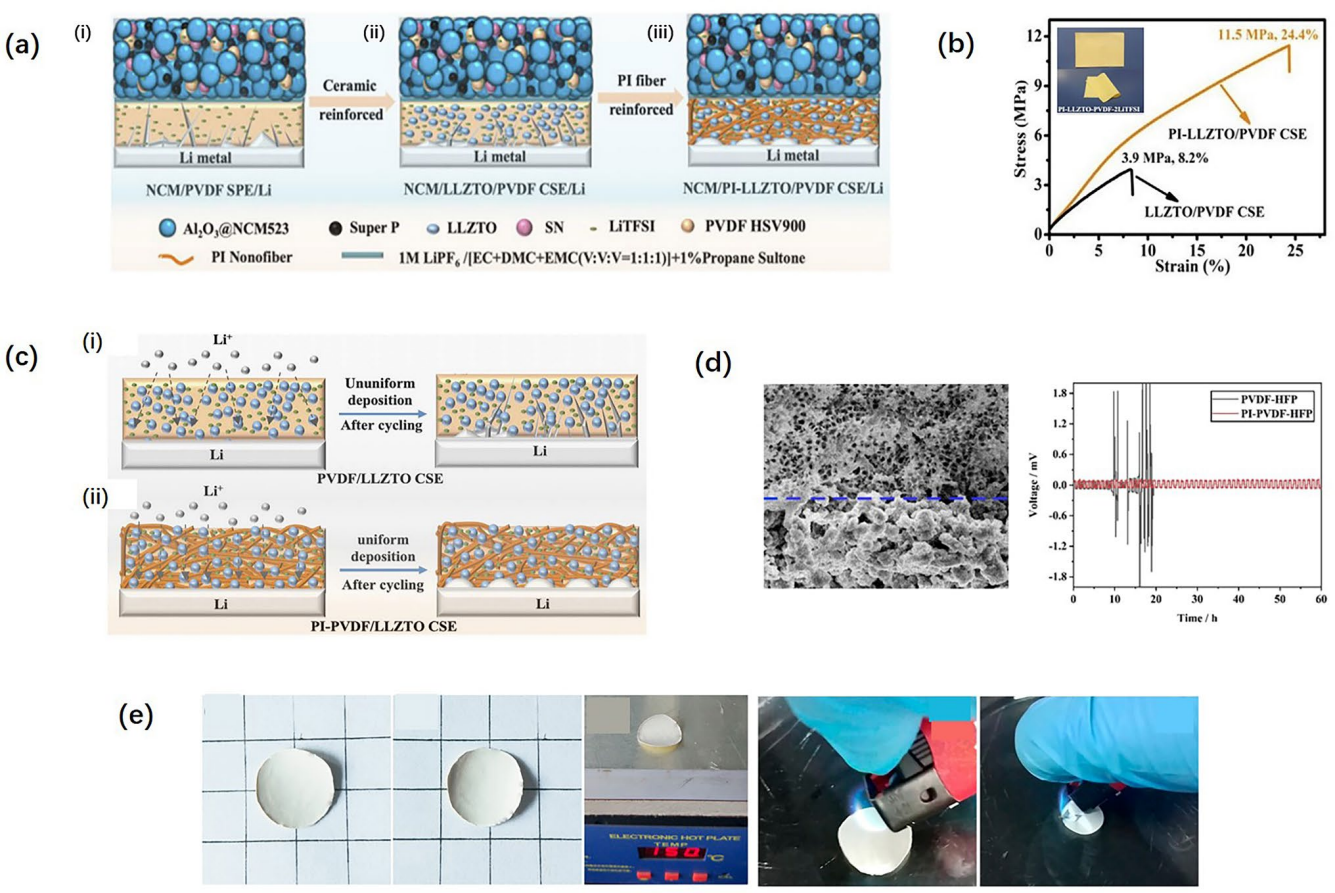
**a** Schematic diagrams of Li-dendrite layers of PVDF SPE (i), LLZTO/PVDF CSE (ii), and PI-LLZTO/PVDF CSE (iii). **b** Mechanical property characterization (inset is the physical morphology). **c** Schematic illustrations of Li-plating/stripping behavior in Li/LLZTO/PVDF CSE/Li (i) and Li/PI-LLZTO/PVDF CSE/Li (ii). **d** SEM image of PI CSE and cycling stability of the symmetric cells assembled with PVDF-HFP and PI-PVDF-HFP CSEs. **e** Thermal stability and flame retardancy of PP and PI films

For solid PI-based CSEs, Hu et al. [88] used the blading method to prepare a PI CSE. The porous PI film was used as the host, and LLZTO nanoparticles with PVDF/LiTFSI were used as electrolyte fillers. The structures of PVDF SPE, LLZTO/PVDF CSE, and PI-LLZTO/PVDF CSE are shown in Fig. 8a(i-iii), where the tensile strength of the PI-LLZTO/PVDF CSE membrane is the best shown in Fig. 8b. The Li-ion plating/stripping mechanism is schematically illustrated in Fig. 8c(i, ii). The result demonstrates that PI CSE exhibits better mechanical strength to resist Li dendrites and forms a more uniform Li deposition layer than the non-PI-based CSEs. Gai et al. [89] prepared an asymmetric PI CSE using the casting method with Li_1.3_Al_0.3_Ti_1.7_(PO_4_)_3_, poly(vinylidene fluoride-hexafluoropropylene) [P(VDF-HFP)], and succinonitrile cast on the PI film. The SEM image of the cross section of the PI-PVDF-HFP CSE is exhibited in Fig. 8d. The porous structure is beneficial to Li-ion conductivity. The Li||Li^+^ coin cell assembled with the PI-PVDF-HFP CSE presents better cycling performance than PVDF-HFP CSE, demonstrating that PI materials are good solid-state electrolyte hosts. The PI-PVDF-HFP CSE is thermally stable at 150 °C and is flame retardant, which is described in Fig. 8e. Notably, the suitable processing methods combined with the outstanding properties of PIs make solid PI-based electrolytes promising SSE candidates in all-solid-state LIBs.

**Fig. 9:**
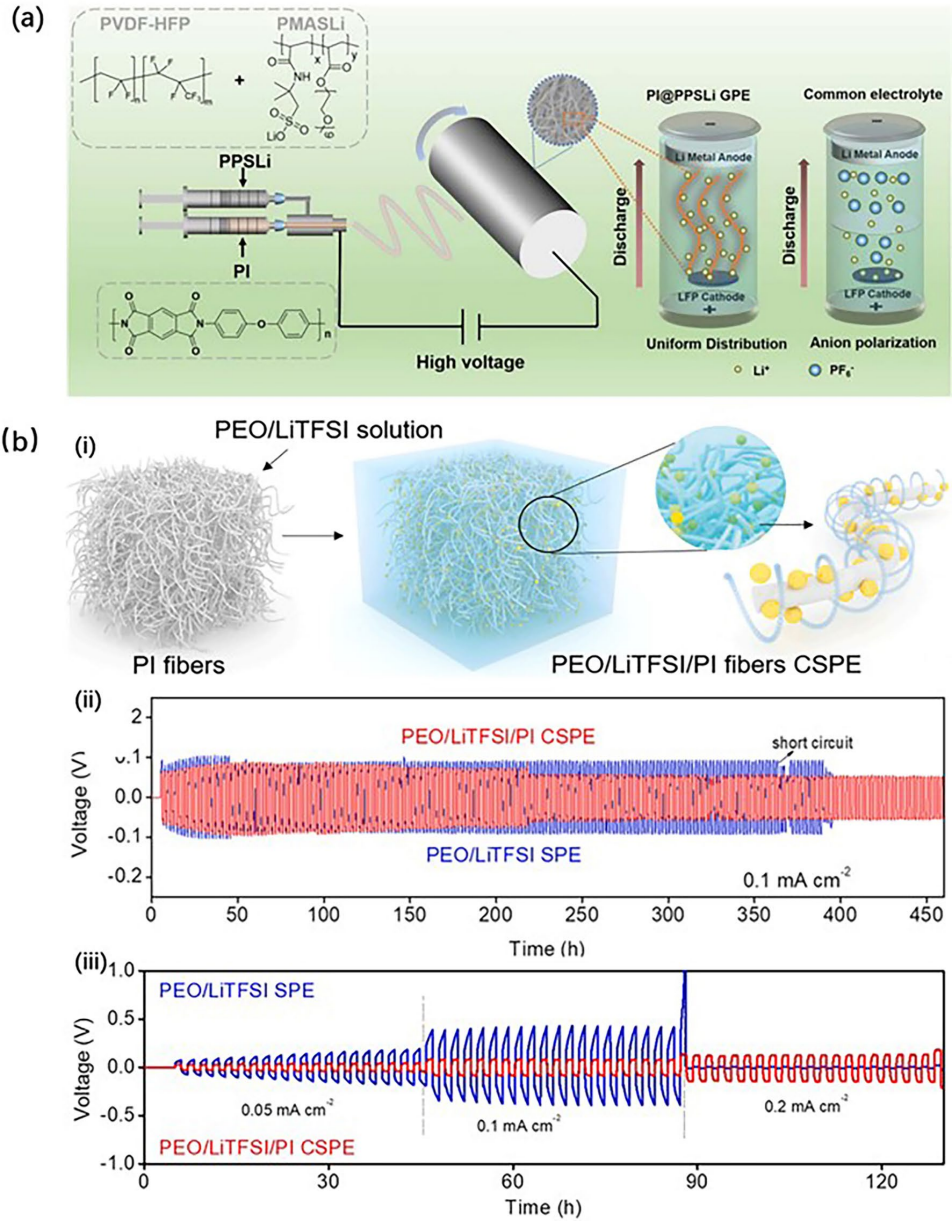
**a** Preparation process of the core–shell gel polymer electrolyte by the coaxial spinning technology. **b** (i) Schematic sketch of PEO/LiTFSI/PI fibers CSPE, (ii) cycling performance of Li|PEO/LiTFSI/PI SPE|Li battery at 0.1 mA cm^−2^, and (iii) rate performance of Li|PEO/LiTFSI/PI SPE|Li battery at different current densities

Electrospinning technology can also be used to prepare the scaffold of solid-state electrolytes. Shen et al. [90] take the electrospinning technology to make core–shell structured gel polymer electrolytes, whose preparation process is shown in Fig. 9a. In detail, the electrolyte solution PVDF-HFP/PMASLi and PI solution were injected into the coaxial nozzle, spun onto the collector wrapped with Al foil, then took it off, and carried on the post-treatment, obtaining the PI-based solid-state electrolytes. Another preparation of PI-based solid-state electrolytes [91] is to make a PI electrospinning membrane, then blend it with PEO/LiTFSI electrolyte solution, and obtain the PI-base solid-state electrolytes, as shown in Fig. 9b. Due to its high mechanical strength, good thermal stability, and excellent (electro)chemical inertness, the cell assembled as Li|PEO/LiTFSI/PI SPE|Li showed better cycling and rate performance than the common cell of Li|PEO/LiTFSI SPE|Li. It follows that the preparation methods of PI separators can be effectively used as reference to prepare PI-based electrolytes.

### Next-generation of Organic Electrodes: PI Active Storage Materials

In LIBs, active storage materials play a crucial role: lithium-ion storage. Based on the different lithium-ion storage mechanisms of cathodes and anodes, active storage materials can be classified into two kinds: cathode and anode. Cathode active storage materials are mainly lithium-contained transition metal oxides, while anode active storage materials are commonly using graphite. Conventional cathode active storage materials are price costive and resource-scarce, limiting their large-scale energy storage applications in long term. Furthermore, the transition metal elements of cathode active storage materials are toxic and environmentally polluting, which recycles difficult, requires tedious procedures, and consumes a lot of energy. Compared with cathode active storage materials, the reserves of graphite are abundant. But the flammability and the goal of reducing carbon emissions force us to search for other alternative materials, such as silicon. Therefore, developing renewable and sustainable materials as active storage materials for LIBs is critical.

The root of lithium-ion storage for organic materials is based on the reversible lithium-ion combination reactions. Presently, many different organic functional groups such as organosulfur compounds [92, 93], nitroxyl radical-bearing compounds [94, 95], conjugated carbonyl-containing compounds [96], imine-functionalized compounds [97, 98], azo-functionalized compounds [99, 100], and newly appeared sulfonamides compounds [101–103] have been used as cathode materials in LIBs. Nevertheless, several small organic molecules can dissolve into the electrolyte, further affecting cycling stability. One of the most effective strategies is to polymerize small active molecules into polymers that are insoluble in electrolytes. Among various polymeric systems, polyimides (PIs), a class of organic carbonyl polymers, seem to be one of the promising electrode materials owing to their satisfying capacity, excellent cycling performance, and good rate capability. Moreover, they are structurally adjustable, safe when fully charged, and environmentally friendly.

In addition, another lithium-ion storage mechanism is like graphite by introducing large amounts of aromatic structures. This mechanism has been found by many references [104–106], and they utilize the conjugated benzenes to store lithium ions.

**Fig. 10:**
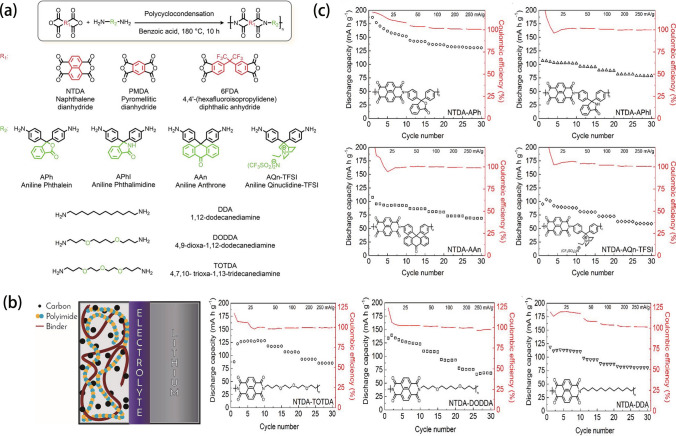
**a** Polycyclocondensation reaction mechanism and chemical structures of starting monomers. **b** Schematic of LIB assembled with polyimide cathode. **c** Rate capability performance

Similarly, PI active storage materials can also be divided into the cathode and anode, two types. As the cathode active storage materials, the lithium-ion storage mechanism is depending on the reversible reduction reactions of carbonyls in PI repeat units. This reaction mainly takes place at 1.5 ~ 3.5 V. Presently, the most widely used dianhydrides are pyromellitic dianhydrides (PMDA), naphthalene-tetracarboxylic dianhydrides (NTCDA), and perylene-tetracarboxylic dianhydrides (PTCDA). Much effort has been devoted to exploring the relationship between PI structure and electrochemical performances. Hernández et al. [107] synthesized many different PI active storage materials by the polycondensations of PMDA, NTCDA, and 4,4’-(hexafluoroisopropylidene)diphthalic anhydride (6FDA) with various diamines shown in Fig. 10a. They utilized these PI active storage materials to assemble LIBs, whose structure is schematically shown in Fig. 10b. As NTCDA-PIs possess good conjugation structure, it shows better cycling performance and rate capability than PMDA-PIs and 6FDA-PIs. Then, NTCDA was used as the main dianhydride to synthesize a series of PI active storage materials, whose rate capability performances are shown in Fig. 10c. Firstly, NTCDA was polymerized with different aromatic diamines such as 3,3-bis-(4-aminophenyl)phthalide (APh), 3,3-bis-(4-aminophenyl)phthalimidine (APhl), 9,9-Bis-(4-aminophenyl)anthrone (AAn), and 3,3-bis(4-aminophenyl)quinuclidine) (AQn-LFSI), where NTCDA-APh PIs exhibit the best rate capability (146 mAh g^−1^ at 25 mA g^−1^, 100% of its theoretical capacity), as it took place the three-electron reaction where two electrons were from the imide part and the additional one from the extra carbonyl group of diamine. NTCDA-AAn PIs only take place the two-electron reactions (96 mAh g^−1^ at 25 mA g^−1^, 73% of its theoretical capacity) as the extra carbonyl group was not redox-active. Similarly, NTCDA-APhl PIs also take place the two-electron reactions (107 mAh g^−1^ at 25 mA g^−1^, 73% of its theoretical capacity) as the extra amide moiety was not redox-active. Additionally, when the ionic group is introduced in the structure of PIs as NTDA-AQn-TFSI, it can deliver the specific capacity of 90 mAh g^−1^, suggesting that the ionic moiety is not able to improve the rate capability of the polymer. They also investigated PIs synthesized with different aliphatic diamines including 4,7,10-trioxa-1,13-tridecanediamine (TOTDA), 4,9-dioxa-1,12-dodecanediamine (DODDA), and 1,12-dodecanediamine (DDA) and found that the NTCDA-DODDA PIs and NTCDA-TOTDA PIs exhibit better rate capability than NTCDA-DDA PIs due to the presence of oxyethylene fragments which is known to solvate lithium ions and improve the ionic conductivity. Therefore, the conjugation structure, redox-active carbonyl group, and oxyethylene fragments are three kinds of important structural units to tune the electrochemical performance.

**Fig. 11:**
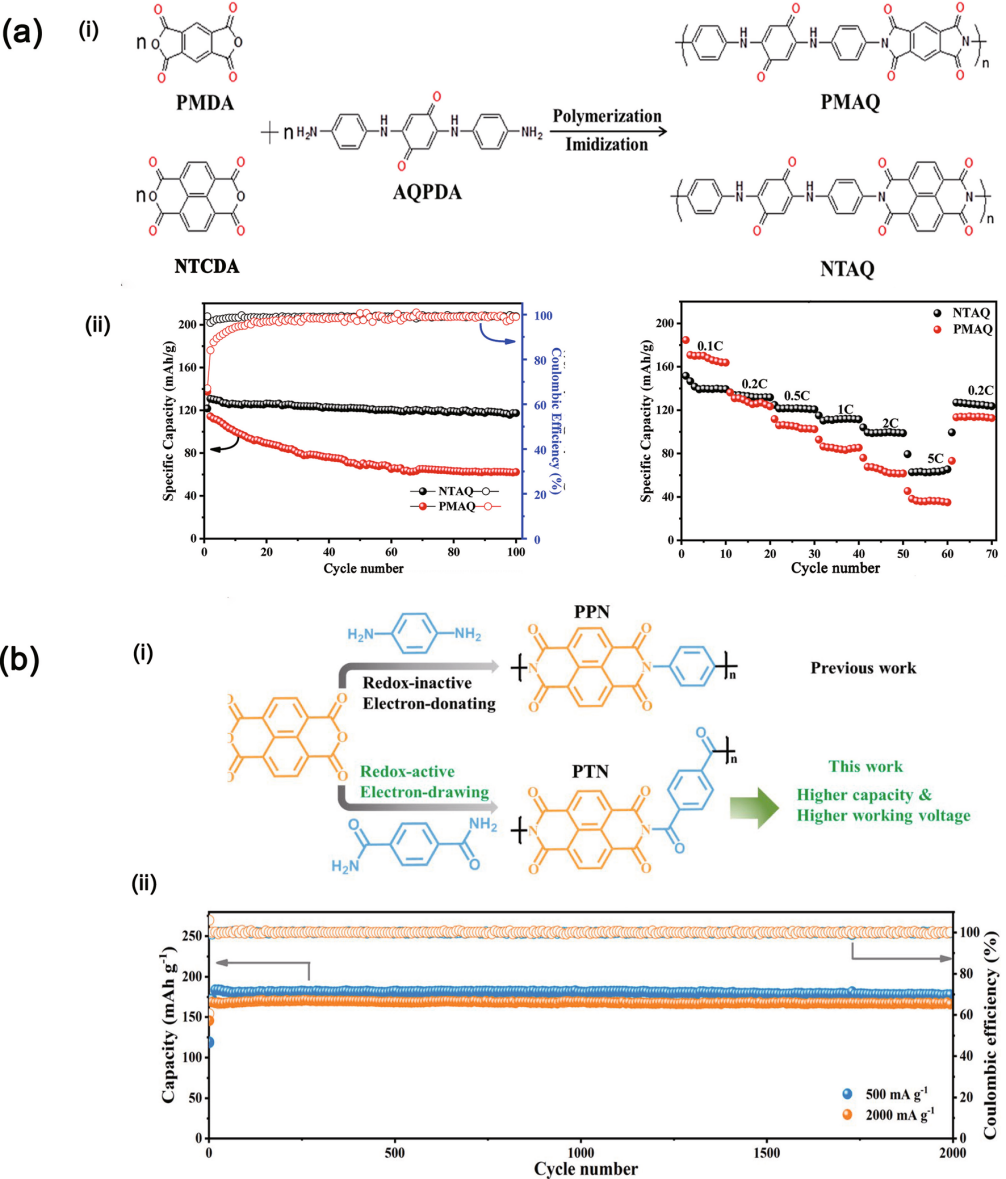
**a** Structures and synthesis of PMAQ and NTAQ (i) as well as the electrochemical performance (ii) and **b** structure of PTN (i) and its good cycling performance (ii)

Moreover, using redox-active anthraquinone units is also an effective strategy to design conjugated carbonyl polymers. Ba et al. [108] prepared the PMDA and NTCDA styles of PIs with another new diamine monomer containing a benzoquinone unit (AQPDA) shown in Fig. 11a. The specific capacity of these two PI cathodes at 0.1 C is 170 and 145 mAh g^−1^, respectively. The reason that PIs can be used as active materials is that active carbonyl groups introduced in molecular structures are more easily combined with lithium ions, playing the role of delivering lithium ions. Wang et al. [109] used a novel conjugated diketone to react with NTCDA to obtain a kind of multi-carbonyl PI cathode active storage material that shows a very stable cycling performance as shown in Fig. 11b. This PI cathode can deliver the specific capacity of 213 mAh g^−1^ (utilization of active sites is 78.7%) and the maximum energy density of 490 Wh kg^−1^, higher than traditional LIBs (300 Wh kg^−1^), demonstrating the significant application value.

**Fig. 12:**
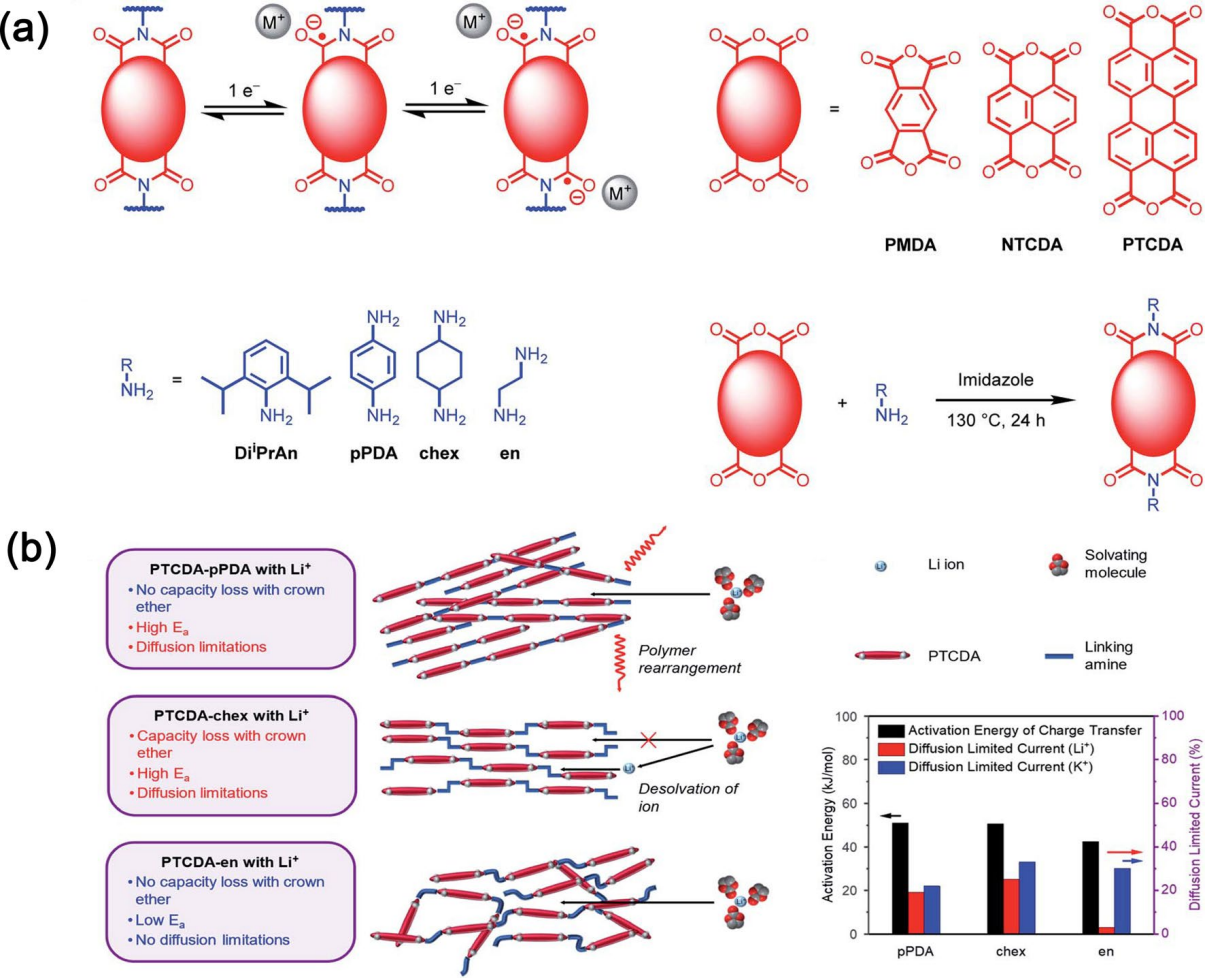
**a** Two one-electron reductions of diimides and the synthesis process of polyimides by tetracarboxylic acid dianhydrides and amine. **b** The charge compensation mechanisms for PTCDA-pPDA, PTCDA-chex, and PTCDA-en as cathodes in LIBs as well as CV tests of activation energy of charge transfer with Li-ion and diffusion-limited current proportion in Li^+^ and K^+^ batteries for the PDI-based polymers

Additionally, the stacking structure of polyimides is important in determining electrochemical performance. Gannett et al. [110] used the strongest conjugation structure of PTCDA to react with para-phenylenediamine (pPDA), trans-1,4-diaminocyclohexane (chex), and 1,2-ethylenediamine (en) to obtain polyimides with different crystallinity, flexibility, and stacking mode shown in Fig. 12a. The results demonstrate that PTCDA-en possesses rapid Li-ion conductivity due to alkyl linking units endow that with an amorphous structure. PTCDA-en possesses the smallest activation energy of charge transfer and diffusion-limited current shown in Fig. 12b, demonstrating that suitable packing structure should be also considered.

**Fig. 13:**
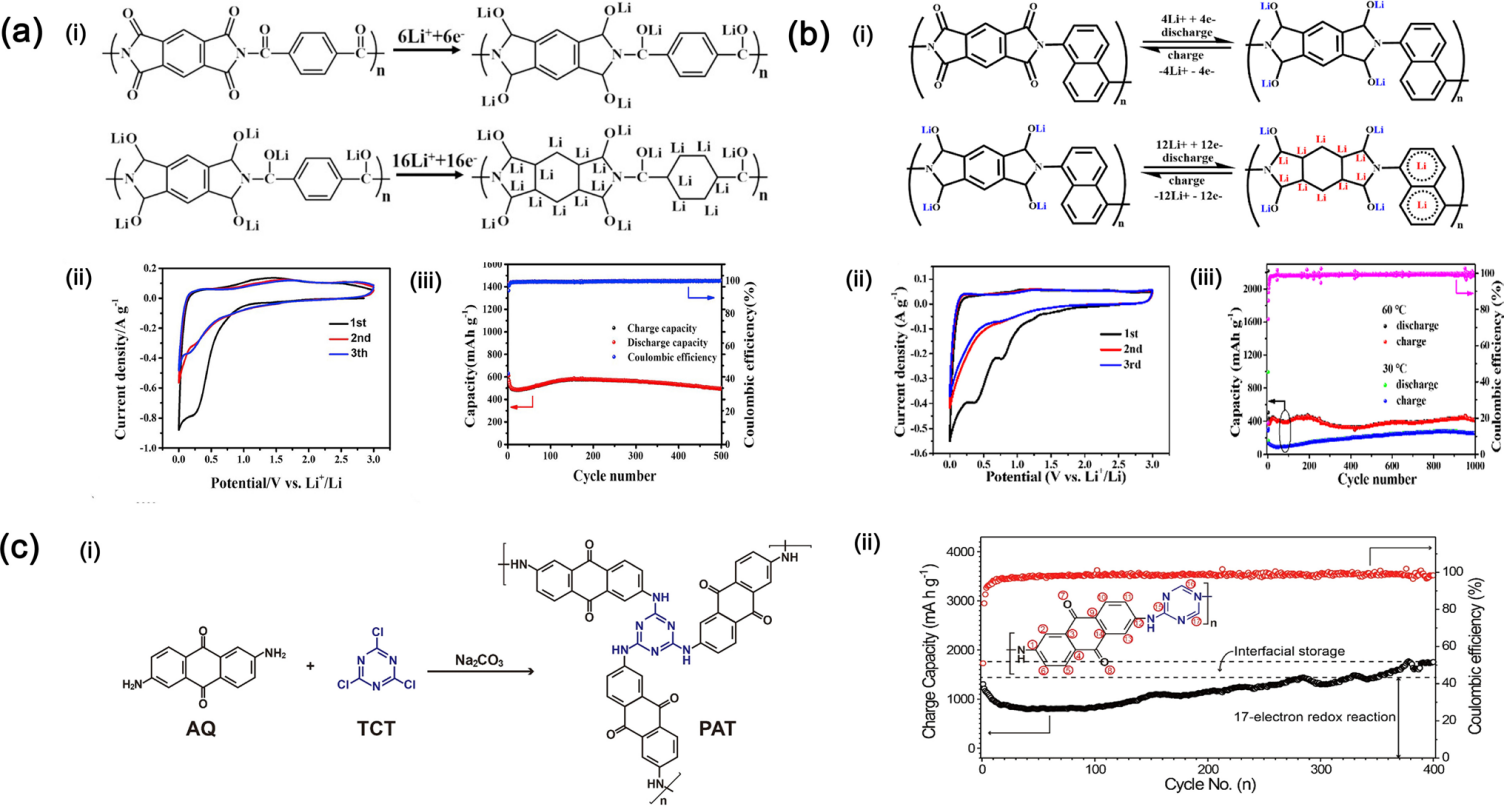
**a, b** Lithiation mechanism (i); (ii) CV test results; (iii) cycling performance. **c** Synthesis process of PAT (i) and cycling performance (ii)

When PI active storage materials are used as anodes, the lithium-ion storage mechanism is mainly attributed to the “hyperlithiation” phenomenon. The suitable operating voltage is at 0 ~ 1 V. More benzenes introduced to the PI repeat unit are beneficial to lithium-ion storage. As shown in Fig. 13a (i), He et al. [111] used PMDA and terephthalate (TA) to synthesize the PI active storage materials with six carbonyl groups per repeat unit. The as-prepared PI materials can be lithiated with 22 lithium ions, displaying the theoretical specific capacity of 1704 mAhg^−1^. CV test results shown in Fig. 13a (ii) confirmed the existence of enolization reactions of carbonyls and the “hyperlithiation” phenomenon. In the cycling process shown in Fig. 13a (iii), the specific capacity will increase, indicating the “hyperlithiation” process has taken place and the specific capacity reaches the maximum value. After this extremum, the specific capacity began to decrease. Changing the repeat units of PIs, the reversible carbonyl reduction reactions and the “hyperlithiation” process will occur, whose process is also trapped by the work of Liao et al. [112] in Fig. 13b(i-iii). Moreover, the ordered and conjugated structure of PIs, such as covalent organic framework, can form many lithium-ion storage channels and accommodate 17 lithium ions per repeating unit during the lithiation process, further enhancing the cycling performance [113] shown in Fig. 13c (i, ii).

**Fig. 14:**
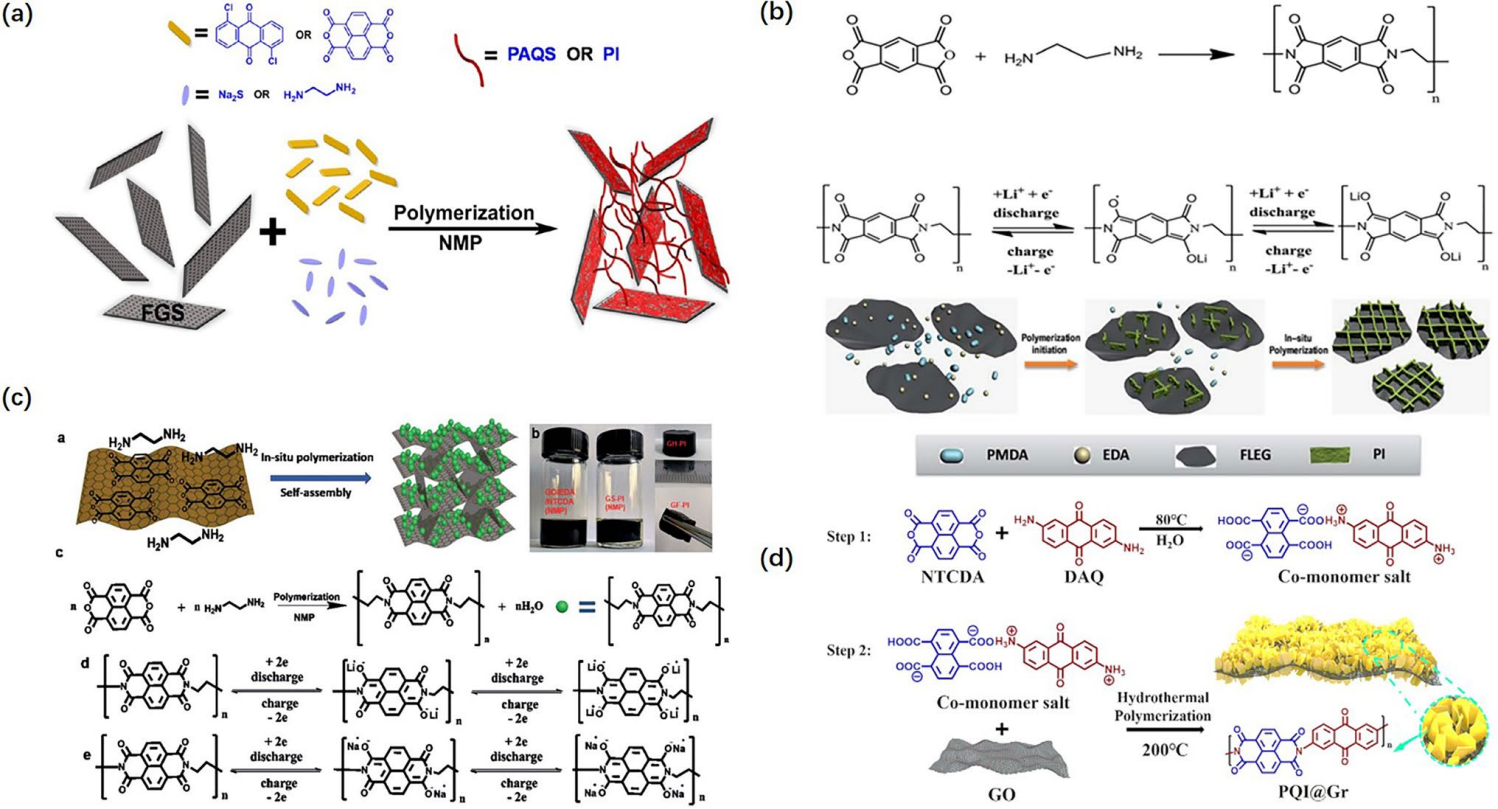
**a** In situ polymerization process of PAQS-FGS or PI-FGS nanocomposite. **b** Preparation process of GF-PI. **c** Synthesis of PI-FLEG via in situ polymerization and Li-ion storage mechanism. **d** Synthetic route of PQI@Gr composites

To further improve the cycling and rate capacity, different kinds of conducting materials, such as graphene, carbon nanotubes, and carbon black, were compounded with PI active storage materials to prepare electrodes. Much effort is utilizing graphene to synthesize the PI-Graphene electrode materials by the *in situ* polymerization strategy, which could make full use of the surface area of graphene and possess several magnitudes higher electronic conductivity compared to pure PI polymers. In Fig. 14a, the graphene and two kinds of carbonyl polymers poly(anthraquinonyl) sulfide (PAQS) and polyimide (PI) were blended to prepare graphene/PI nanocomposites by the in situ polymerization method [98]. In Fig. 14b, the monolithic 3D graphene/polyimide composites (GF-PI) were prepared by the one-step solvothermal strategy [114]. In Fig. 14c, Ahmad et al. [115] developed a novel PI-FLEG (few-layer exfoliated graphene) nanocomposite, where PI nano-flakes shorten the diffusion path length between the electrolyte and electrode interfaces, leading to a further increase in the rate capability. In Fig. 14d, NTCDA and 2,6-diamino-anthraquinone (DAQ) as redox-active monomers in the presence of graphene oxide (GO) were in situ polymerized to obtain the graphene-sandwiched PQI nanosheets (PQI@Gr) [116]. Obviously, graphene is a promising conductive material.

**Fig. 15:**
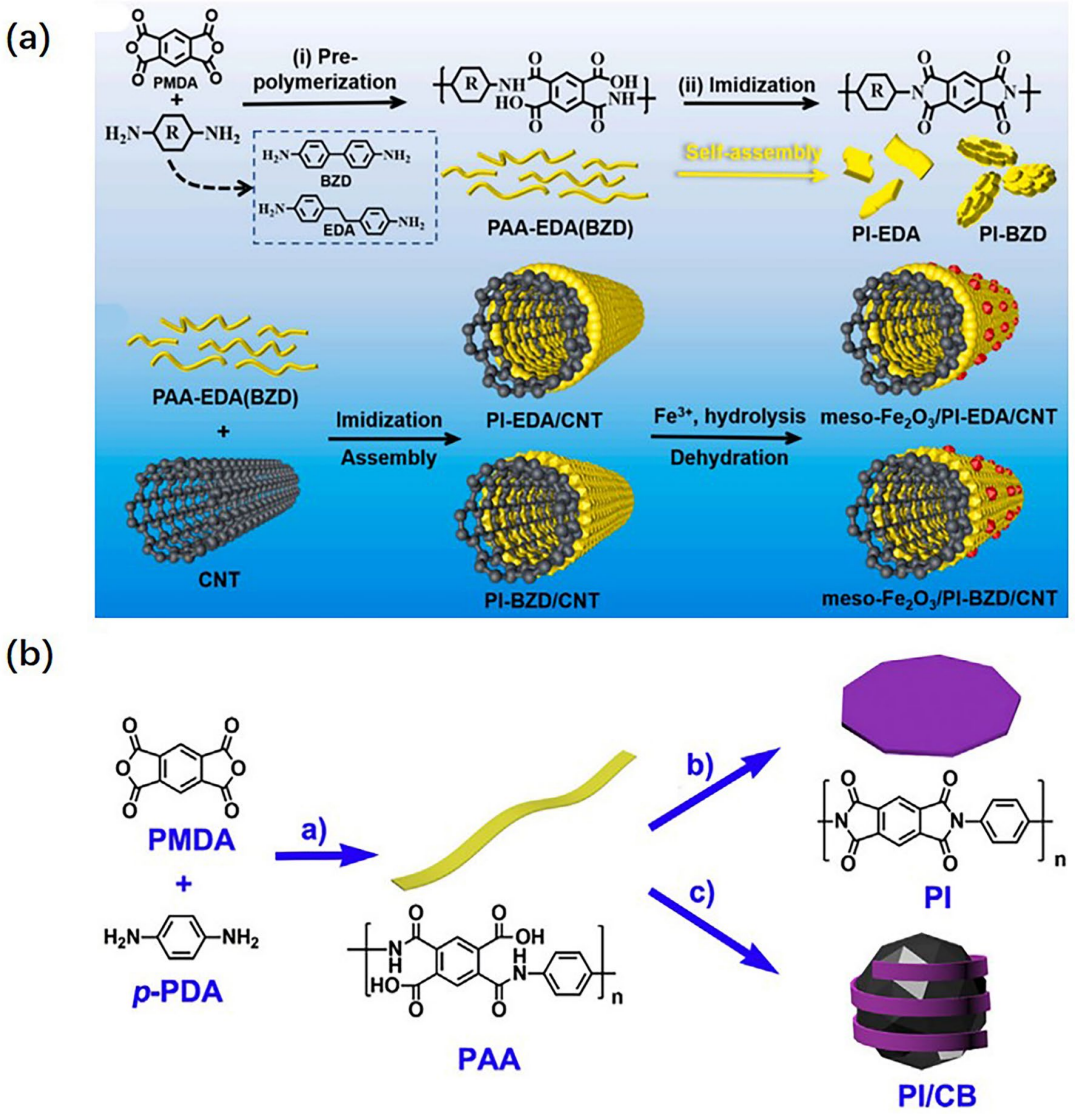
**a** Schematic illustration of the synthetic strategy of meso-Fe_2_O_3_/PI/CNT. **b** The illustration of the in situ polymerization process of the PI–CB composites

Apart from graphene, carbon nanotubes and carbon black are also reported to be used in the preparation of active storage materials. Combining a sequential assembly and high-temperature dehydration strategies, carbon nanotube (CNT)/polyimide (PI)/mesoporous Fe_2_O_3_ (meso-Fe_2_O_3_/PI/CNT) ternary nanocomposite materials were prepared by Ban et al. [117] as shown in Fig. 15a. Zhang et al. [118] fabricated the composites of polyimide and carbon black (PI/CBs) by the *in situ* condensations of PMDA and pPDA with the presence of carbon black shown in Fig. 15b. Due to the high conductive property of carbon materials, the capacity of PI active storage materials can be effectively utilized, further increasing the energy density. Currently, increasing the specific capacity and operating voltage are two effective strategies to increase energy density. Nevertheless, the average operating voltage is limited by the intrinsic structures of PI molecules, which are less than 3.5 V usually. Designing structures that can withstand high voltage is challenging. Therefore, increasing the specific capacity is the first choice. The effective method is by introducing more carbonyls or conjugated structures which are beneficial to increase the lithium-ion storage capacity and further increase the energy density.

## Perspectives on Using PIs in Practical LIBs

**Fig. 16:**
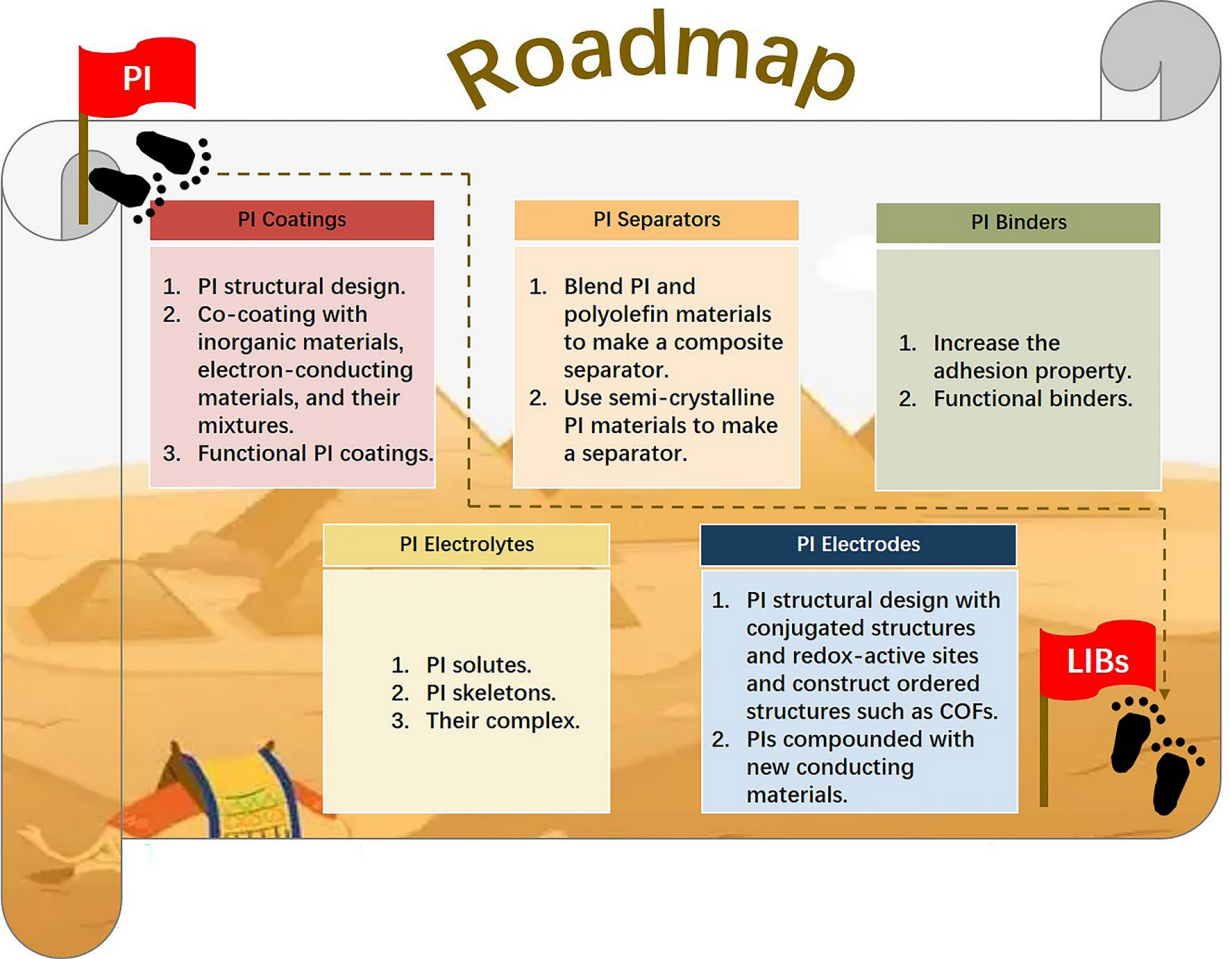
Strategies of PIs applied in LIBs

PIs have many advantages, including excellent mechanical properties, outstanding thermal stability, satisfactory electrolyte wettability, and good (electro)chemical resistance, which are promising candidates for coatings, separators, binders, solid-state electrolytes, and active storage materials. Appropriate PIs need to be selected based on application requirements. This section summarizes the main issues that PIs can address in current LIBs and proposes strategies for researchers in developing PIs for LIBs. Figure 16 lists the strategies for PIs applied in LIBs.

### PI Coatings

The coating is an effective way to improve the performance of high-voltage cathodes. To date, only a few studies have reported PI coatings, even though PIs have some advantages over traditional inorganic materials. The outstanding features of PIs such as designable molecular structures, continuous texture, and high-voltage/high-temperature resistance all attract research interest.

**Fig. 17:**
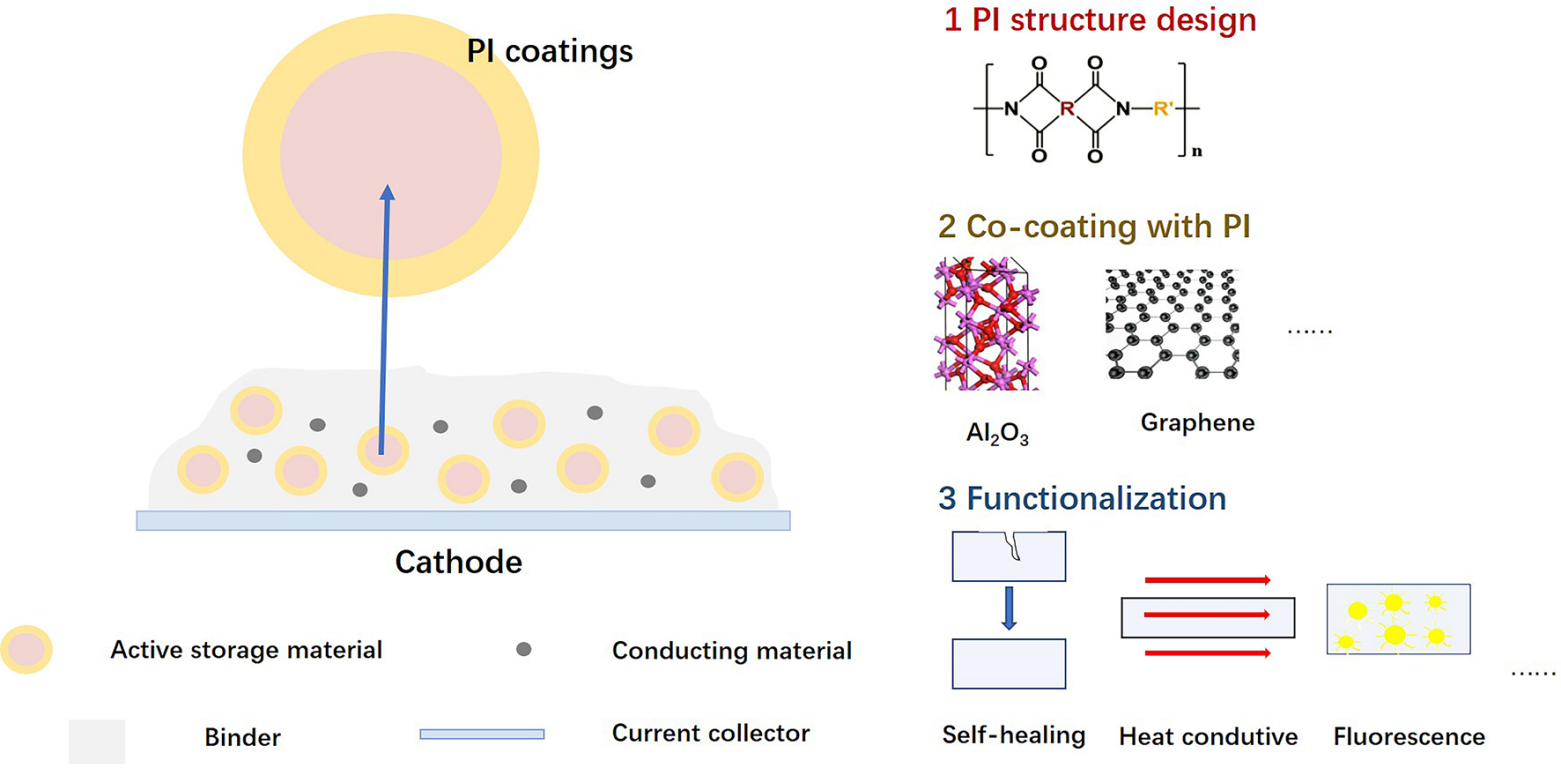
Design strategies of PI coatings

PI coatings can be implemented in three ways. First, researchers could use monomers with different structures to synthesize PI coatings with targeted performance. Second, to improve the electrochemical performance, inorganic materials (such as Al_2_O_3_, ZrO_2_, and TiO_2_), electron-conducting materials (such as graphene, carbon nanotubes, and carbon black), and their mixtures can be compounded with PIs to achieve co-coating layers [119, 120]. Third, self-healing, heat conductive, fluorescence, etc., functions can be introduced to PIs to realize the functionalization of cathode materials. Figure 17 shows the design strategies of PI coatings.

Moreover, PI coatings are not only limited to cathodes, but they can also coat silicon anodes [121]. These all deserve to explore further.

### PI Separators

The separators play a vital role in the safety of LIBs. Once the separator is destroyed due to thermal shrinkage, ISCs may happen, causing thermal runaway. Therefore, LIBs have strict requirements for separator performance. First, the separator should possess high mechanical strength to separate the cathode and anode under high tensile and compressive stress. Second, the separator should be thermally stable with little or limited thermal shrinkage (< 5%). Further, the separator needs to be wettable by the electrolyte. Moreover, its pore distribution should be uniform to allow lithium ions to pass through. Finally, the separator should be highly insulating. Although polyethylene separators have been commercialized, their high thermal shrinkage and low trigger temperatures cause poor safety in LIBs. Currently, there are three ways to enhance the thermal stability of polyolefin separators: (1) using PP/PE/PP composite separators; (2) coating polyolefin separators with thermally stable inorganics; and (3) coating polyolefin separators with thermally stable polymers. However, these approaches still cannot satisfy the requirements for safe batteries.

**Fig. 18:**
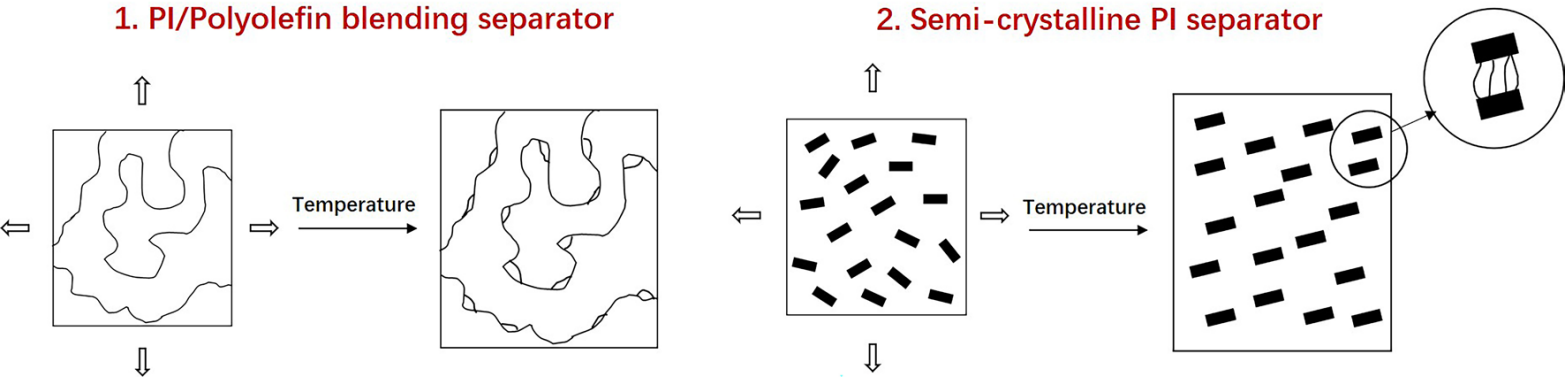
Processing techniques for PI separators

The PI separators can improve safety in LIBs. However, PIs have only been used in research laboratories due to three reasons: They absorb water and are expensive and difficult to process. Choosing hydrophobic monomers or designing hydrophobic structures can address the water absorption issue. To decrease costs, researchers should devote effort to reducing raw material costs and improving processing equipment. The most critical issue in processing is to achieve uniform pore distribution. Two strategies to solve the issue of pore-making can be followed. The first is by blending PI and polyolefin materials to obtain a composite separator. This approach utilizes the good processability of polyolefins to achieve uniform and well-distributed pores during heating and stretching. PIs contribute to electrolyte wettability, mechanical strength, and thermal stability. Second, a pure semi-crystalline PI can be designed and prepared. The separator can be obtained by stretching and orienting at its melting point. These approaches all require improvements in the preparation capability of the processing equipment, especially at high temperatures. Figure 18 shows the processing techniques for PI separators.

### PI Binders

Large volume expansion and shrinkage during cycling in silicon anodes cause rapid decay of electrochemical performance. Elastic binders can constrain silicon particles to avoid pulverization and electrical contact loss. However, PI binders with 3D crosslinking networks can maintain the anode integrity while accommodating high silicon active material content. The adhesive properties of the anode also need to be enhanced to prevent silicon anode pulverization. Enhancement could be achieved by introducing adhesive functional groups such as hydrogen bonds or metal–ligand coordination interactions in the binder. Besides these conventional approaches, multi-functionalization can also be one of the future directions for PI binders. For example, electronic or Li-ion conducting functional groups can be introduced to compensate for the limited electronic or ionic conduction. Safety and lifespan should also be considered. In summary, functional binders are a crucial research topic.

**Fig. 19:**
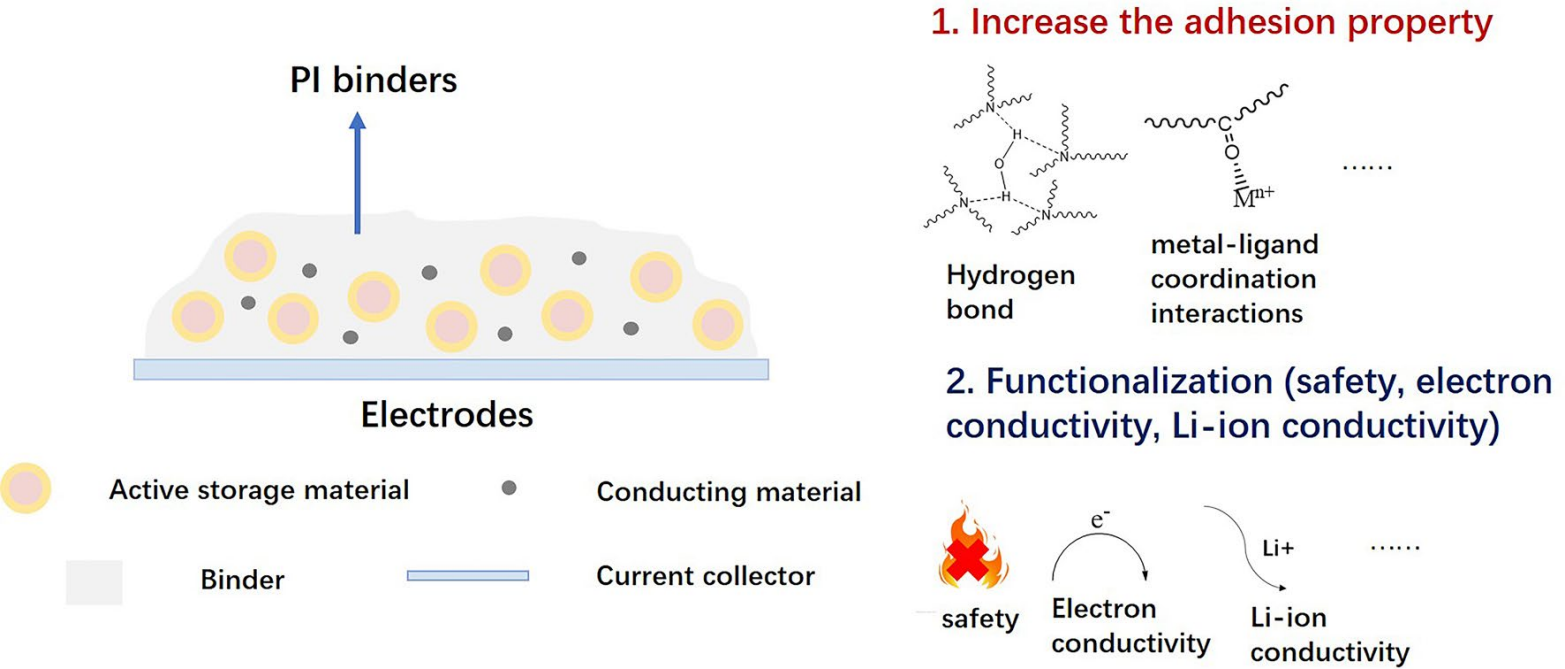
Design strategies of PI binders

In fact, there are some references reported on PI binders for cathodes [79, 80, 122–124]. Several important references [80, 122–124] are from the group of Qi et al. to explore high-voltage performance. The above-modified strategies are also fit for PI cathode binders. Figure 19 shows the design strategies of PI binders.

### PI Solid-State Electrolytes

**Fig. 20:**
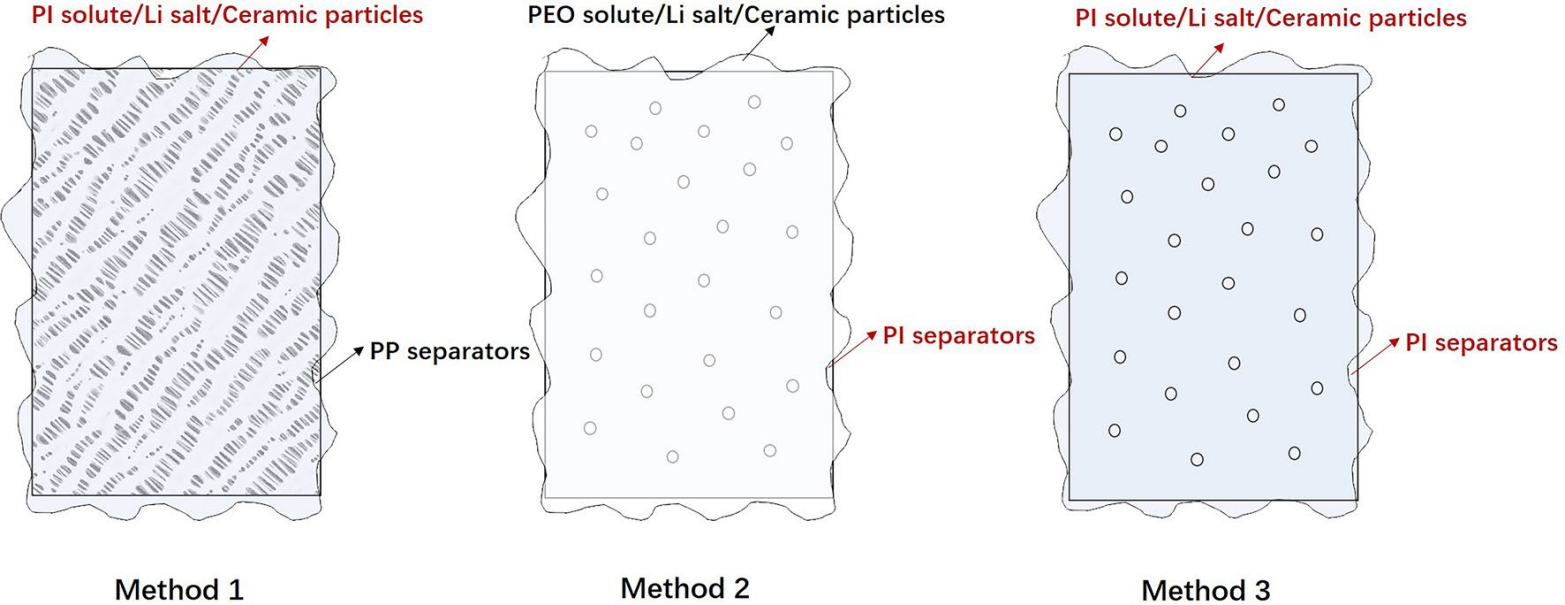
Design strategies of PI SSEs

The PI-based SSEs may be essential in all-solid-state LIBs because they can function as a separator and an electrolyte. Apart from this, there are three methods for designing PI SSEs. First, traditional separators can be coated with PI solutes and lithium salts or ceramic particles. Second, PI itself can be used as the separator matrix with other polymer solutes, lithium salts, and ceramic particles coated on its surface. Third, PI separator is coated with PI solutes and lithium salts or ceramic particles. Figure 20 shows the design strategies of PI SSEs.

### PIs Active Storage Materials

**Fig. 21:**
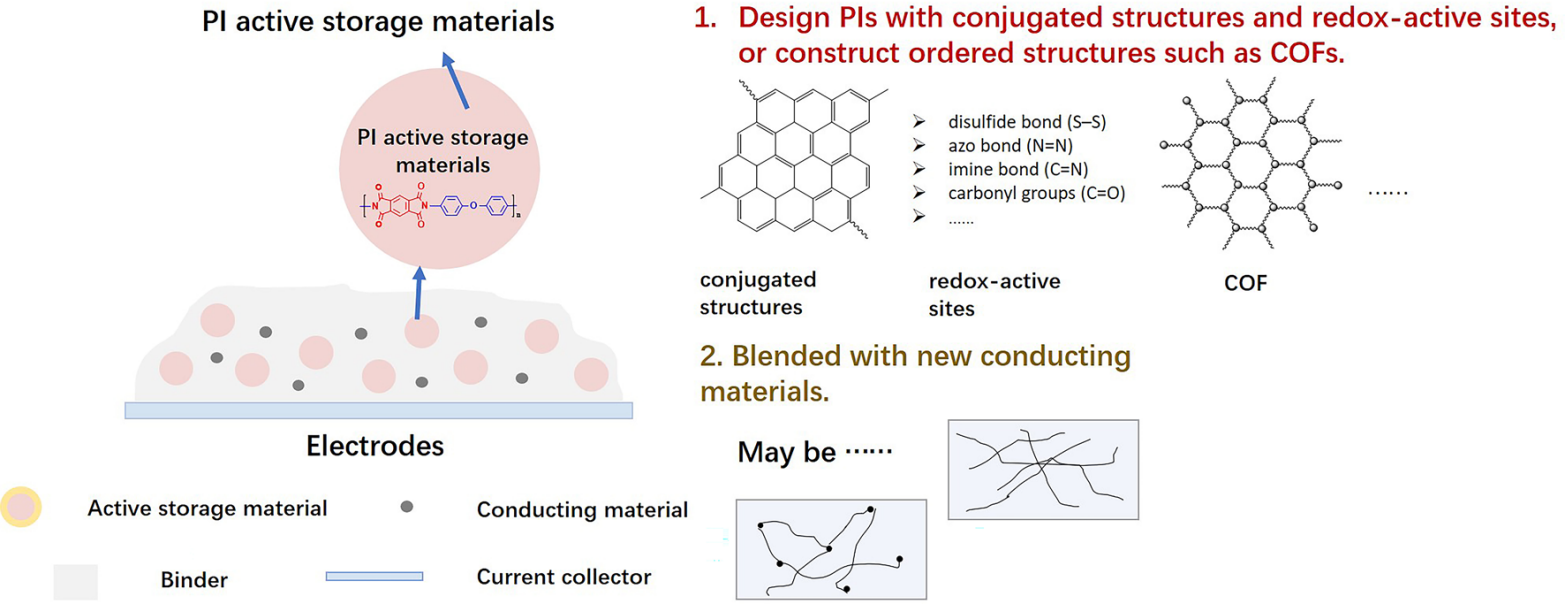
Design strategies of PI active storage materials

In recent years, a variety of organic active storage materials have developed rapidly, among which PIs with stable structures, large theoretical capacity, good heat resistance, high tensile modulus, and high safety have attracted wide attention. However, there are still some problems in the practical application of PI active storage materials including the low utilization rate of redox-active carbonyl groups and poor electronic conductivity. To solve these problems, the following two strategies are adopted. Firstly, the reasonable structure design of PI active storage materials should be promoted. The energy storage mechanism of active storage materials is closely related to the conjugated structures and redox-active sites. Utilizing the large π–π packing structures is encouraged, and more redox-active functional groups should be introduced such as the disulfide bond (S–S), azo bond (N = N), and imine bond (C = N) besides carbonyl groups (C = O). Furthermore, ordered structures such as popular covalent organic framework compounds (COFs) linked by imine units should be considered. Secondly, more advancing electron-conducting materials should be investigated and introduced to the PI active storage materials to enhance the energy utilization rate of PIs. Figure 21 shows the design strategies of PI active storage materials.

## Outlook and Perspective

In this perspective, we presented the recent progress in PI coatings, separators, binders, SSEs, and active storage materials, revealing the potential of PIs in improving LIB safety and electrochemical performance. We identified several strategies to further improve LIBs using PIs. In summary, future works on implementing PIs in LIBs should consider the following three points:

1. Devoting much effort to designing the PI molecular structure and choosing suitable preparation methods for critical PI components according to LIB requirements.
2. Advancing functional PI and PI composites to decrease cost and improve LIB energy density, rate capacity, cycling stability, and safety.
3. Developing practical processing technologies and accelerating industrialization to implement PI components in LIBs.

Promoting the development of new PI materials and taking advantage of performance benefits from these new materials advances LIBs. Next-generation LIBs can be economical, environmentally benign, and safe to use. Meanwhile, large-scale LIBs utilization can relieve the energy crisis. Hence, high-performance LIBs could advance our connected world toward a more sustainable future.
